# Reliability of the NIH toolbox cognitive battery in children and adolescents: a 3-year longitudinal examination

**DOI:** 10.1017/S0033291720003487

**Published:** 2022-07

**Authors:** Brittany K. Taylor, Michaela R. Frenzel, Jacob A. Eastman, Alex I. Wiesman, Yu-Ping Wang, Vince D. Calhoun, Julia M. Stephen, Tony W. Wilson

**Affiliations:** 1 Department of Neurological Sciences, Cognitive Neuroscience of Development & Aging (CoNDA) Center, and the Center for Magnetoencephalography, University of Nebraska Medical Center, Omaha, NE, USA; 2 Department of Biomedical Engineering, Tulane University, New Orleans, LA, USA; 3 Mind Research Network, Albuquerque, NM, USA

**Keywords:** Child and adolescent development, cognitive neuroscience, longitudinal, NIH toolbox, test-retest reliability

## Abstract

**Background:**

The Cognitive Battery of the National Institutes of Health Toolbox (NIH-TB) is a collection of assessments that have been adapted and normed for administration across the lifespan and is increasingly used in large-scale population-level research. However, despite increasing adoption in longitudinal investigations of neurocognitive development, and growing recommendations that the Toolbox be used in clinical applications, little is known about the long-term temporal stability of the NIH-TB, particularly in youth.

**Methods:**

The present study examined the long-term temporal reliability of the NIH-TB in a large cohort of youth (9–15 years-old) recruited across two data collection sites. Participants were invited to complete testing annually for 3 years.

**Results:**

Reliability was generally low-to-moderate, with intraclass correlation coefficients ranging between 0.31 and 0.76 for the full sample. There were multiple significant differences between sites, with one site generally exhibiting stronger temporal stability than the other.

**Conclusions:**

Reliability of the NIH-TB Cognitive Battery was lower than expected given early work examining shorter test-retest intervals. Moreover, there were very few instances of tests meeting stability requirements for use in research; none of the tests exhibited adequate reliability for use in clinical applications. Reliability is paramount to establishing the validity of the tool, thus the constructs assessed by the NIH-TB may vary over time in youth. We recommend further refinement of the NIH-TB Cognitive Battery and its norming procedures for children before further adoption as a neuropsychological assessment. We also urge researchers who have already employed the NIH-TB in their studies to interpret their results with caution.

The National Institutes of Health (NIH), in collaboration with over 200 field-specific experts, developed the NIH Toolbox (NIH-TB), which is an assessment of cognitive, motor, and emotional functioning that is standardized for persons 3–85 years of age (Beaumont et al., 2013; Weintraub et al., 2013a, b). Of particular interest to researchers and clinicians, the NIH-TB Cognitive Battery was designed to measure abilities within the domains of working (Tulsky et al., 2013) and episodic memory (Bauer et al., 2013), attention and executive functioning (Zelazo et al., 2013), processing speed (Carlozzi, Tulsky, Kail, & Beaumont, 2013), and language (Gershon et al., 2013), and was created to be efficacious for assessing longitudinal outcomes in research studies (http://www.healthmeasures.net/explore-measurement-systems/nih-toolbox). The Cognitive Battery was validated against gold-standard neuropsychological assessments and showed good short-term (7 to 21 days) test-retest reliability in adults and children (Heaton et al., 2014; Mungas et al., 2014; Weintraub et al., 2013a, b). Perhaps unsurprisingly, the NIH-TB has garnered wide-spread interest in the scientific community and has been adopted as a primary tool for assessing cognitive abilities in numerous investigations, including large-scale longitudinal projects like the Adolescent Brain and Cognitive Development (ABCD) study (Luciana et al., 2018). In its early release, experts cautioned that the NIH-TB Cognitive Battery was not designed to assess neuropsychological status or injury, and had not been tested for its sensitivity to neuropsychological disparities (Bauer & Zelazo, 2013; Beaumont et al., 2013).

In response to this potential shortcoming, researchers developed fully normed T-scores for the NIH-TB Cognitive Battery using a normative sample of children and adults (Casaletto et al., 2015). Specifically, the fully normed T-scores correct for a swath of demographic factors known to impact and potentially bias scores on neuropsychological assessments, including age, sex, ethnicity, race, and educational attainment (for children, the NIH-TB uses the mother's level of education). The original report demonstrating the fully normed T-scores notes that, of all the standardized scores available from the Toolbox, including uncorrected and age-corrected standard scores, the T-scores are the best indicator of potential individual impairment and could be used to determine deviations from previous levels of neurocognitive functioning (Casaletto et al., 2015). Several studies have since examined the potential utility of the NIH-TB's fully normed T-scores in clinical populations, including individuals with neurological disorders (Carlozzi et al., 2017) and persons with social anxiety (Troller-Renfree, Barker, Pine, & Fox, 2015). Expressing high confidence, the NIH-TB manual for scoring and interpretation now explicitly states that the fully normed T-scores, ‘are primarily intended for neuropsychological applications…’ (NIH, 2016, p. 2)

Despite rapidly growing interest in utilizing the NIH-TB Cognitive Battery for neuropsychological assessments (Tulsky & Heinemann, 2017), including by our own team, research has yet to establish the longer-term stability of these fully normed T-score metrics of cognitive performance. As mentioned previously, studies have shown good short-term test-retest reliability of the NIH-TB Cognitive Battery assessments with a retest period of 7–21 days (Weintraub et al., 2013a, b), However, retesting individuals over longer intervals to determine treatment effects lasting longer than 21 days, and/or gains and losses over time would also be of major interest to both clinicians and scientists (Harvey, 2012). This matter is of exceptional importance given the growing adoption of the NIH-TB in large-scale longitudinal studies aiming to examine the development of cognitive abilities across the lifespan, often testing participants only on an annual basis.

The purpose of the present study was to determine the long-term temporal stability of the fully normed T-scores from the NIH-TB Cognitive Battery in children and adolescents (ages 9–15 years-old). For completeness and proper comparison, we also assessed the stability of the uncorrected and the age-corrected standardized scores. All participants were enrolled at one of two sites, with near equal enrollment at each site, and were invited to return annually for 3 years. Thus, we were able to examine test-retest reliability of the NIH-TB Cognitive Battery at three intervals: (1) from Year 1 to Year 2, (2) from Year 2 to Year 3, and (3) from Year 1 to Year 3. We determined temporal stability metrics (e.g. intraclass correlation coefficients; ICCs) for the full sample, and separately for each site. Analyses were conducted for each of the seven tests comprising the NIH-TB Cognitive Battery, as well as the three composite scores (crystalized, fluid, and total composite scores) yielded by the assessment. Based on prior literature, good test-retest reliability was determined based on the lower bound of the 95% confidence interval (CI) for each ICC, where >0.70 is acceptable for research use, and >0.90 is acceptable for clinical use (Aldridge, Dovey, & Wade, 2017; Drost, 2011; Streiner, Norman, & Cairney, 2015). We hypothesized that, based on prior short-term reliability studies, the NIH-TB measures would have good test-retest reliability among samples recruited from both sites. In particular, the fully normed T-scores should express strong reliability given the strict norming procedures employed and thus were expected to account for maturational changes over time, and to be robust to any demographic differences in samples participating at the two sites.

## Methods

### Participants

A total of 212 typically-developing children and adolescents were recruited across two sites during Year 1 of the National Science Foundation-funded Developmental Chronnecto-Genomics (Dev-CoG) study (http://devcog.mrn.org/). Participants had no diagnosed neurological, psychiatric, or developmental disorders and no history of head trauma or substance use disorder. Additionally, no participants were using medications that might alter neural functioning. Participants were between 9 and 15 years-of-age at the time of their first visit (*M*_site 1_ = 11.75 years, *s.d.* = 1.79; *M*_site 2_ = 11.80 years, *s.d.* = 1.87), and all participants were invited to return annually for 3 years (time between visits 1 and 2: *M*_site 1_ = 1.09 years, *s.d.* = 0.16; *M*_site 2_ = 1.16 years, *s.d.* = 0.23; time between visits 2 and 3: *M*_site 1_ = 1.02 years, *s.d.* = 0.084; *M*_site 2_ = 1.13 years, *s.d.* = 0.32). All demographic data were reported by a parent or legal guardian as part of the intake process during Year 1. Parents of the child participants signed informed consent forms, and child participants signed assent forms before proceeding with the study. All procedures were approved by the appropriate Institutional Review Board for each site.

### NIH-TB cognitive battery

All participants completed the NIH-TB Cognitive Battery on a tablet during each year of the study. All tests were administered in a fixed order per instructions in the manual. It took approximately 1 hour to complete the battery each year. Each data collection site had trained research assistants who administered the computerized protocol in accordance with the manual and ensured participants' compliance and understanding throughout the testing process. The Cognitive Battery of the NIH-TB consists of seven assessments, each purporting to measure a different cognitive construct. Briefly, *Dimensional Change Card Sort* (*DCCS*) is a measure of executive functioning; the *Flanker test* assesses attention and inhibitory control; *List Sorting Working Memory* (*WM*) assesses working memory abilities; *Pattern Comparison* measures processing speed; *Picture Sequence Memory* is an indicator of episodic memory; *Oral Reading* assesses reading and language abilities; and *Picture Vocabulary* assesses vocabulary comprehension. Three composite scores are derived from the subtests: *Crystallized Cognition*, *Fluid Cognition*, and *Total Cognition*. Complete details of each Cognitive Battery test are reported in prior work (Heaton et al., 2014; Mungas et al., 2013, 2014; Weintraub et al., 2013a, b). Following standard procedure, the fully normed T-scores were calculated within the NIH-TB software and used for further analyses. According to prior literature, these T-scores were normed for age, sex, ethnicity, race, and mother's educational attainment, and should result in a mean of 50 with a standard deviation of 10 (Casaletto et al., 2015; NIH, 2016). Additionally, both uncorrected and age-corrected standard scores were also extracted from the NIH-TB software; reliability indices for these scores are reported in Supplemental Materials.

### Data analysis

We assessed the long-term test-retest reliability of each NIH-TB Cognitive Battery assessment using multiple indices of stability, including concordance correlation coefficients (CCCs), root mean squared differences (RMSDs), Pearson correlations, and intraclass correlation coefficients (ICCs). For CCCs and RMSDs, we calculated both consistency (C,1) and absolute agreement (A,1) measures (Barchard, 2012; Scott, Sorrell, & Benitez, 2019). With respect to ICCs, we calculated two-way mixed-effects models [ICC(3,1)] of both consistency and agreement (Berchtold, 2016; Koo & Li, 2016; Stratford, 1989; Streiner et al., 2015; Weir, 2005). Based on prior literature, good test-retest reliability was determined based on the lower bound of the 95% CI for each ICC, where >0.70 is acceptable for research use, and >0.90 is acceptable for clinical use (Aldridge et al., 2017; Drost, 2011; Streiner et al., 2015). Finally, we compared reliability metrics for each NIH-TB Cognitive Battery test between sites using Fisher's *r* to *Z* transformations of Pearson correlation coefficients for each of the three periods of interest. Analyses were performed in SPSS version 26.

## Results

### Descriptives

Of 212 recruited participants, 192 reported complete demographic data necessary for calculating fully normed T-scores during Year 1. Demographic data for the final sample are reported in Table 1, separately for each site and each year of data collection. Overall, participants were relatively well matched demographically across sites with one notable exception; Site 2 had a larger proportion of participants who identified as Hispanic/Latino compared to Site 1 (Year 1: χ^2^(1) = 37.98, *p* < 0.001). Regardless, fully normed T-scores obtained from the NIH-TB Cognitive Battery should be robust in controlling for demographic differences between participant samples. Means and standard deviations of the T-scores for each test, by site and year, are detailed in Table 2. Means were generally near or slightly above the expected mean of 50, with the exception of the Flanker test where average fully normed T-scores were about 0.5 s.d. below 50 and consistently lower than all other test scores across both sites. However, overall, group means for each year were relatively consistent, with only minor shifts in the group averaged T-scores over time.

**Table 1.** Participant demographic distributions for the total sample and separately by site for each year of data collection for participants included in reliability analyses

		Year 1						Year 2						Year 3					
		Total sample		Site 1		Site 2		Total sample		Site 1		Site 2		Total sample		Site 1		Site 2	
		*N*	%	*N*	%	*N*	%	*N*	%	*N*	%	*N*	%	*N*	%	*N*	%	*N*	%
	*N* Total	192		100		92		162		83		79		118		65		53	
Sex	Male	98	51.04	50	50.00	49	53.26	84	51.85	42	50.60	42	53.16	68	57.63	37	56.92	31	58.49
	Female	94	48.96	51	51.00	43	46.74	78	48.15	41	49.40	37	46.84	50	42.37	28	43.08	22	41.51
Race	White	169	88.02	90	90.00	80	86.96	149	91.98	78	93.98	71	89.87	106	89.83	60	92.31	46	86.79
	Black or African American	5	2.60	3	3.00	2	2.17	2	1.23	0	0	2	2.53	2	1.69	0	0	2	3.77
	Asian	0	0	0	0	0	0	0	0	0	0	0	0	0	0	0	0	0	0
	American Indian/Alaska Native	5	2.60	0	0	5	5.43	5	3.09	0	0	5	6.33	4	3.39	0	0	4	7.55
	Multiracial	13	6.77	8	8.00	5	5.43	6	3.70	5	6.02	1	1.27	6	5.08	5	7.69	1	1.89
Ethnicity	Hispanic or Latino	39	20.31	3	3.00	36	39.13	32	19.75	3	3.61	29	36.71	24	20.34	2	3.08	22	41.51
	Not Hispanic or Latino	153	79.69	98	98.00	56	60.87	130	80.25	80	96.39	50	63.29	94	79.66	63	96.92	31	58.49
First Language	English	185	96.35	100	100.00	85	92.39	156	96.30	83	100.00	73	92.41	113	95.76	65	100.00	48	90.57
	Spanish	4	2.08	0	0	4	4.35	3	1.85	0	0	3	3.80	3	2.54	0	0	3	5.66
	Other	3	1.56	0	0	3	3.26	3	1.85	0	0	3	3.80	2	1.69	0	0	2	3.77
Second Language	None	165	85.94	97	97.00	68	73.91	139	85.80	81	97.59	58	73.42	102	86.44	64	98.46	38	71.70
	Spanish	8	4.17	1	1.00	7	7.61	7	4.32	1	1.20	6	7.59	5	4.24	0	0	5	9.43
	English	7	3.65	0	0	7	7.61	5	3.09	0	0	5	6.33	3	2.54	0	0	3	5.66
	Other	8	4.17	1	1.00	7	7.61	7	4.32	0	0	7	8.86	4	3.39	0	0	4	7.55
	(No Response)	4	2.08	1	1.00	3	3.26	4	2.47	1	1.20	3	3.80	4	3.39	1	1.54	3	5.66
Mother's Education	Less than 7th Grade	3	1.56	1	1.00	2	2.17	3	1.85	1	1.20	2	2.53	2	1.69	1	1.54	1	1.89
	Junior High/Middle School	2	1.04	0	0	2	2.17	2	1.23	0	0	2	2.53	2	1.69	0	0	2	3.77
	Partial High School	2	1.04	0	0	2	2.17	2	1.23	0	0	1	1.27	1	0.85	0	0	1	1.89
	High School Graduate	4	2.08	0	0	4	4.35	4	2.47	0	0	4	5.06	4	3.39	0	0	4	7.55
	Partial College	24	12.50	12	12.00	12	13.04	23	14.20	12	14.46	11	13.92	15	12.71	10	15.38	5	9.43
	College Education	73	38.02	43	43.00	30	32.61	57	35.19	34	40.96	23	29.11	41	34.75	26	40.00	15	28.30
	Graduate Degree	84	43.75	44	44.00	40	43.48	57	35.19	36	43.37	36	45.57	53	44.92	28	43.08	25	47.17

Note: The demographics table above does not report characteristics of participants who were excluded from the present study (*N* = 20) due to missing demographic data necessary for calculating fully normed T-scores for the NIH Toolbox measures.

**Table 2.** Means and standard deviations of fully normed *T-*scores for each NIH Toolbox Cognitive Battery assessment fully normed *T*-score by year, for the full sample and separately for each site

		Year 1			Year 2			Year 3		
		All	Site 1	Site 2	All	Site 1	Site 2	All	Site 1	Site 2
DCCS	*M*	50.92	51.28	50.45	49.43	51.34	47.06	50.40	52.44	47.57
	*s.d.*	10.70	11.67	9.33	11.47	11.94	10.47	13.06	13.99	11.19
Flanker	*M*	45.61	45.84	45.30	43.36	43.31	43.42	45.05	46.13	43.55
	*s.d.*	9.62	10.20	8.87	8.68	9.21	8.04	9.29	9.98	8.09
ListWM	*M*	52.87	53.01	52.68	53.68	52.57	55.06	54.15	53.28	55.37
	*s.d.*	10.38	10.71	9.99	10.02	9.89	10.07	8.63	8.56	8.67
ProcSpeed	*M*	51.09	55.91	44.73	54.43	56.95	51.31	57.23	59.43	54.18
	*s.d.*	14.97	14.21	13.57	14.44	14.42	13.95	13.66	14.05	12.62
PicMem	*M*	52.56	54.60	49.88	56.84	57.16	56.45	56.91	59.31	53.52
	*s.d.*	11.67	12.28	10.27	11.88	11.98	11.82	12.93	13.09	12.03
OralRead	*M*	52.31	54.19	49.86	53.39	54.24	52.34	53.30	54.28	51.92
	*s.d.*	11.02	11.60	9.75	10.89	11.71	9.77	10.12	10.11	10.07
PicVocab	*M*	54.55	54.00	55.27	55.80	55.80	55.80	55.47	55.38	55.59
	*s.d.*	10.99	10.94	11.08	11.76	11.44	11.44	10.20	10.16	10.37
Crystal	*M*	53.99	54.59	53.21	55.38	55.76	54.90	55.94	56.66	51.55
	*s.d.*	10.44	10.51	10.36	10.87	11.31	10.36	11.60	12.99	10.62
Fluid	*M*	50.67	52.98	47.66	52.31	53.38	51.00	54.47	56.66	51.55
	*s.d.*	10.69	11.39	8.89	11.86	12.56	10.86	12.25	12.99	10.62
Total	*M*	52.69	54.51	50.30	54.54	55.45	53.41	55.94	57.46	53.92
	*s.d.*	10.59	11.00	9.58	11.34	12.17	10.20	11.60	12.64	9.79

Note: Total sample (‘All’): *n_year1_* = 192, *n_year2_* = 162, *n_year3_* = 118; Site 1: *n_year1_* = 100, *n_year2_* = 83, *n_year3_* = 65. Site 2: *n_year1_* = 92, *n_year2_* = 79, *n_year3_* = 53. DCCS = Dimensional Change Card Sorting; Flanker = Flanker Test of Inhibitory Control and Attention; ListWM = List Sorting Working Memory; ProcSpeed = Pattern Comparison Processing Speed; PicMem = Picture Sequence Memory; OralRead = Oral Reading; PicVocab = Picture Vocabulary; Crystal = Crystalized Cognition composite score; Fluid = Fluid Cognition composite score; Total = Total Cognition composite score.

### Test-Retest reliability

**Fig. 1.**
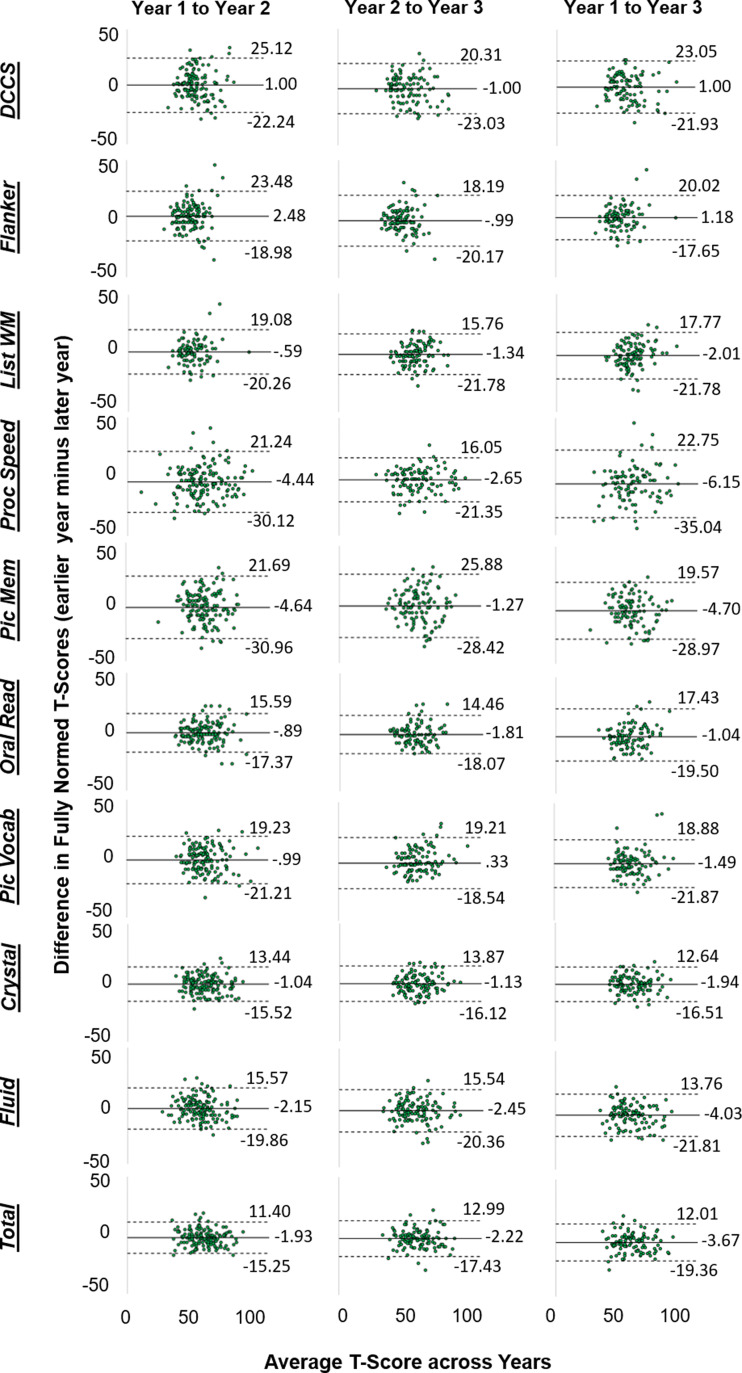
Bland-Altman plots depicting patterns of deviation in fully normed T-scores over time for the full sample, collapsed across study sites. The solid black line in each plot is the bias (i.e. the mean difference between years); dashed lines are the upper and lower limits of agreement (bias ± 1.96*s.d.). DCCS = Dimensional Change Card Sorting; Flanker = Flanker Test of Inhibitory Control and Attention; List WM = List Sorting Working Memory; Proc Speed = Pattern Comparison Processing Speed; Pic Mem = Picture Sequence Memory; Oral Read = Oral Reading; Pic Vocab = Picture Vocabulary; Crystal = Crystalized Cognition composite score; Fluid = Fluid Cognition composite score; Total = Total Cognition composite score.

**Fig. 2.**
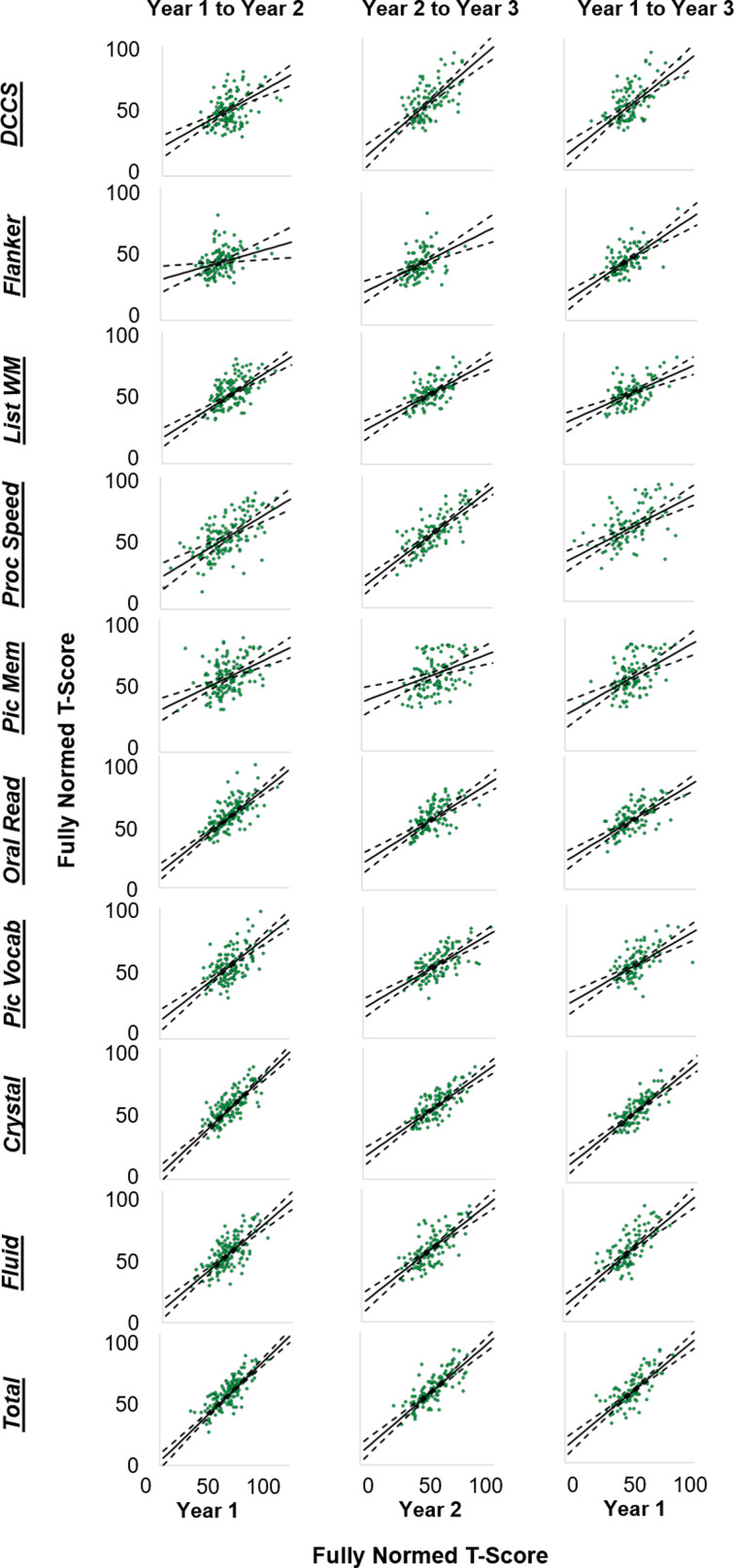
Scatterplots depicting the correlations between fully normed T-scores for each NIH-TB Cognitive Battery subtest, collapsed across study sites, for each of the three tested intervals. Solid black lines indicate the line of best fit (i.e. the Pearson correlation) through the data. Dashed black lines show the upper and lower bounds of the 95% confidence interval around the line of best fit. DCCS = Dimensional Change Card Sorting; Flanker = Flanker Test of Inhibitory Control and Attention; List WM = List Sorting Working Memory; Proc Speed = Pattern Comparison Processing Speed; Pic Mem = Picture Sequence Memory; Oral Read = Oral Reading; Pic Vocab = Picture Vocabulary; Crystal = Crystalized Cognition composite score; Fluid = Fluid Cognition composite score; Total = Total Cognition composite score.

As demonstrated in Figs 1 and 2, there was substantial individual variability in patterns of change over time (for site-specific data, see online Supplementary Figs. S1-S2). Pearson *r*, and consistency and absolute agreement CCCs and ICCs generally exhibited good agreement, with similar conclusions drawn about stability regardless of the reliability metric utilized (Table 3). Examination of the absolute agreement ICCs indicated a broad range of stability estimates, with coefficients ranging between 0.31 and 0.76 for the full sample collapsed across sites (Table 3, online Supplementary Table 1). Reliability tended to be better between Year 2 and Year 3 compared to reliabilities between other time points. Most importantly, the lower bound of the 95% CI for each ICC suggested that only three of the tests within the NIH-TB Cognitive Battery met reliability criteria for use in research, though results were mixed across sites and time points (e.g. see *Pattern Comparison Processing Speed* from Year 2 to Year 3, site 1 and site 2; *Crystallized Composite* from Year 1 to Year 3, site 1 only; *Total Composite* from Year 1 to Year 2, site 1 only). Importantly, none of the tests met criteria for clinical use in the full sample, nor within either site for any of the time periods (ranges for minimum bounds of 95% CIs: full sample = 0.17–0.66; Site 1 = 0.061–0.74; Site 2 = 0.013–0.74). This is perhaps unsurprising when viewing the Bland-Altman plots (Fig. 2), which demonstrate deviations in fully normed T-scores across years for each test. There were marked disparities in data clustering across tests and time points; for instance, the data for the Flanker test clustered relatively consistently to the left (poorer average scores), whereas the data for Processing Speed were widely variable and did not seem to follow any specific clustering. Moreover, online Supplementary Table 2 describes the number and percentage of participants whose T-scores deviated by more than one standard deviation (10 points) from one year to another; between 14.72% and 77.97% of the sample deviated by at least one standard deviation for a given test and time period.

**Table 3.** Consistency and absolute agreement reliability indices for the NIH Toolbox Cognitive Battery subtests and composite scores for the full sample collapsed across data collection sites

		Consistency				Absolute Agreement			
	*r*	CCC	RMSD	ICC	95% CI	CCC	RMSD	ICC	95% CI
*Dimensional change card sort*									
Y1 to Y2	0.422	0.421	12.065	0.421	0.279, 0.545	0.418	12.109	0.419	0.278, 0.543
Y2 to Y3	0.603	0.598	11.149	0.597	0.465, 0.704	0.594	11.083	0.596	0.464, 0.702
Y1 to Y3	0.583	0.580	11.467	0.580	0.439, 0.693	0.579	11.426	0.582	0.441, 0.694
*Flanker*									
Y1 to Y2	0.324	0.358	10.240	0.322	0.170, 0.459	0.350	10.390	0.314	0.165, 0.450
Y2 to Y3	0.421	0.422	9.787	0.420	0.256, 0.560	0.421	9.765	0.419	0.256, 0.559
Y1 to Y3	0.520	0.565	8.798	0.516	0.363, 0.642	0.563	8.793	0.514	0.362, 0.640
*List sorting working memory*									
Y1 to Y2	0.528	0.564	9.584	0.527	0.401, 0.634	0.562	9.587	0.528	0.402, 0.635
Y2 to Y3	0.560	0.563	8.788	0.555	0.414, 0.670	0.558	8.828	0.551	0.410, 0.667
Y1 to Y3	0.507	0.513	9.863	0.494	0.338, 0.625	0.500	10.063	0.487	0.331, 0.618
*Pattern comparison processing speed*									
Y1 to Y2	0.624	0.636	12.842	0.623	0.514, 0.712	0.607	13.622	0.599	0.462, 0.703
Y2 to Y3	0.767	0.775	9.486	0.767	0.679, 0.833	0.764	9.737	0.755	0.655, 0.827
Y1 to Y3	0.513	0.526	14.439	0.509	0.354, 0.636	0.480	15.755	0.470	0.280, 0.619
*Picture sequence memory*									
Y1 to Y2	0.364	0.371	13.344	0.364	0.216, 0.495	0.343	14.130	0.340	0.183, 0.478
Y2 to Y3	0.403	0.413	13.668	0.402	0.236, 0.545	0.413	13.609	0.402	0.237, 0.545
Y1 to Y3	0.479	0.482	12.350	0.477	0.319, 0.611	0.447	13.207	0.446	0.264, 0.594
*Oral reading*									
Y1 to Y2	0.718	0.718	8.406	0.718	0.630, 0.788	0.716	8.426	0.717	0.629, 0.787
Y2 to Y3	0.655	0.678	8.126	0.655	0.536, 0.748	0.670	8.244	0.646	0.526, 0.742
Y1 to Y3	0.646	0.638	9.417	0.638	0.509, 0.728	0.635	9.430	0.637	0.509, 0.737
*Picture vocabulary*									
Y1 to Y2	0.608	0.605	10.168	0.607	0.495, 0.699	0.602	10.200	0.606	0.494, 0.699
Y2 to Y3	0.636	0.645	9.691	0.528	0.503, 0.728	0.645	9.647	0.630	0.505, 0.729
Y1 to Y3	0.558	0.599	9.632	0.556	0.410, 0.674	0.589	9.772	0.553	0.408, 0.671
*Crystallized composite score*									
Y1 to Y2	0.744	0.770	7.387	0.744	0.663, 0.808	0.766	7.437	0.744	0.663, 0.808
Y2 to Y3	0.747	0.782	7.276	0.746	0.653, 0.817	0.780	7.303	0.744	0.650, 0.815
Y1 to Y3	0.665	0.775	7.194	0.663	0.544, 0.756	0.765	7.346	0.660	0.540, 0.753
*Fluid composite score*									
Y1 to Y2	0.591	0.688	9.031	0.590	0.474, 0.685	0.676	9.252	0.585	0.469, 0.682
Y2 to Y3	0.716	0.744	8.980	0.716	0.614, 0.794	0.734	8.999	0.703	0.591, 0.787
Y1 to Y3	0.612	0.702	9.185	0.612	0.480, 0.717	0.664	9.988	0.590	0.440, 0.706
*Total composite score*									
Y1 to Y2	0.722	0.817	6.805	0.722	0.635, 0.790	0.805	7.054	0.717	0.629, 0.787
Y2 to Y3	0.772	0.800	7.508	0.772	0.687, 0.836	0.792	7.574	0.759	0.660, 0.830
Y1 to Y3	0.626	0.761	8.005	0.624	0.495, 0.726	0.727	8.703	0.608	0.468, 0.717

Note: Year 1 to Year 2 *N* = 162, Year 2 to Year 3 *N* = 118; Year 1 to Year 3 *N* = 118; ‘*r*’ = Pearson correlation; ‘CCC’ = concordance correlation coefficient; ‘RMSD’ = root mean squared difference; ‘ICC’ = intraclass correlation coefficient; ‘95% CI’ = 95% confidence interval about the ICC; ‘Y1 to Y2’ = reliability measured between year 1 and year 2 of the study; ‘Y2 to Y3’ = reliability measured between year 2 and year 3 of the study; ‘Y1 to Y3’ = reliability measured between year 1 and year 3 of the study.

This is also evidenced by the relatively large RMSD values reported in Table 3, many of which are near or above 10 indicating that a large portion of the sample exhibited shifts in scores near or exceeding one full standard deviation. Such large shifts, if meaningful, could be interpreted as extreme gains or deficits in cognitive functioning year-over-year. For example, a *T* score of 50 in a given year falls into the 50th percentile for performance. Jumping to a score of 60 the following year (+1SD) would place that child in the 84th percentile, whereas a falling to a score of 40 the following year (−1SD) would place that child in the 16th percentile for performance (NIH, 2016, p. 38). Such wide variability could have widespread impacts for interpreting changes in cognitive functioning among youth over time.

Reliability metrics for the uncorrected and the age-corrected standardized scores are reported in online Supplementary Tables 3 and 4, respectively. Briefly, uncorrected standardized scores yielded some of the largest ICC absolute agreement estimates, ranging from 0.30 to 0.79; in fact, when comparing reliability metrics between all three types of scores (T-scores, uncorrected, age-corrected) across all combinations of samples and time points, the uncorrected scores had the largest ICC *estimate* in 46.67% of cases (age-corrected: 32.22%; T-score: 21.11%). Unfortunately, these metrics are deceptive. Further examination of the *lower bound of the 95% CI* about the ICC showed that uncorrected standardized scores had the *poorest* reliability in 68.89% of all cases (age-corrected: 24.44%; T-score: 6.67%), with values ranging from −0.09 to 0.63. T-scores tended to have the best reliability as defined by the lower bound of the 95% CI of the ICC and were the only scores stable enough for use in research in select instances.

### Comparison by site, ethnicity, age, and sex

Fisher's *r* to *Z* transformations comparing test-retest correlations are embedded within online Supplementary Tables 1, 3, and 4. Of note, sites significantly differed in the reliability of List Sorting Working Memory T-scores during all three time periods (*p's* < 0.05). Additionally, sites differed in T-score reliability in seven out of the ten tests when examining the longest-term reliability, from Year 1 to Year 3 (all *p*'s < 0.05), with Site 1 showing better test-retest reliability compared to Site 2 in all cases. Bland-Altman plots by site are illustrated in online Supplementary Fig. 1 and indicate significant variability in patterns of score deviations between sites. Scatterplots demonstrating the correlations between T-scores for each of the three retest intervals, separately by site, with plotted CI about the correlation can be viewed in online Supplementary Fig. 2. Notably, many of the children's data points are broadly distributed outside of the 95% CI for the correlations. Given the pattern of differences in the reliability, and the earlier noted difference in ethnic distributions between sites, we hypothesized that the norming procedures for Hispanic/Latino children may be inadequate. However, follow up testing comparing reliability indices between Hispanic/Latino *v.* non-Hispanic/Latino children showed similar reliabilities between ethnic groups in the present sample (see Supplemental Results, online Supplementary Table 5). We additionally explored any potential deviations in reliability related to age at the start of the study and related to sex (see Supplemental Results, online Supplementary Tables 6 and 7).

## Discussion

The present study investigated the long-term test-retest reliability of the NIH-TB Cognitive Battery in a large cohort of children and adolescents enrolled in a two-site study of typical cognitive and brain development. Specifically, we examined stability across 3 years of fully normed T-scores, which are intended for use in neuropsychological assessment (Casaletto et al., 2015; NIH, 2016). We found wide-ranging levels of score stability across the NIH-TB Cognitive Battery measures, with most tests exhibiting only moderate reliability. When comparing reliability metrics between data collection sites, we noted significant differences primarily emerging in the longest-term interval, between Year 1 and Year 3. Most importantly, despite site differences, a select few of the NIH-TB Cognitive Battery tests exhibited strong enough temporal stability for use in research, but none were reliable enough for use in clinical settings according to field standards (Drost, 2011; Streiner et al., 2015). Among the tests meeting criteria for research, results were disparate across time points and were specific to data collection sites. In accordance with prior work (e.g. Watkins and Smith, 2013), the composite scores of the NIH-TB did tend to show stronger reliability than many of the individual subtests, at least for the full sample and for Site 1. However, the composite scores also showed some of the most robust and temporally-persistent site differences in stability (online Supplementary Table 1). Thus, it seems that the composite scores were not immune to site-specific temporal variability over time.

It is also worth noting that the uncorrected and age-corrected standardized scores generally exhibited poorer reliability than the fully normed T-scores, with the lowest metrics observed for uncorrected standardized composite scores between Year 1 and Year 3 (online Supplementary Tables 3 and 4). These results come in direct contrast to recommendations by the NIH manual for scoring and interpretation, which suggests that uncorrected standard scores above all other metrics may be used to monitor an individual's performance over time (NIH, 2016).

The poorer-than-expected temporal stability of the T-scores may indicate inadequate norming, or measurement error due to test administration or other unforeseen factors. However, differences in test administration are unlikely with the NIH-TB because a computer delivers the majority of instructions and administers the tests. Additionally, a trained research assistant remained in the room for the duration of testing to supply any verbal instructions in accordance with the NIH-TB manual and to ensure participant compliance and understanding. That said, we did observe site differences in this study, which could suggest nuances in the administration can affect the results. For instance, research has shown that such wide-ranging factors as participant fatigue (Johnson, Lange, DeLuca, Korn, & Natelson, 1997), administrator-participant rapport (Barnett, Parsons, Reynolds, & Bedford, 2018), and aspects of the testing environment (e.g. lighting, ambient noise, etc.; Gavin, Davies, Schmidt, and Segalowitz, 2008) can contribute to an individual's performance during neuropsychological testing. With so much room for variability, and thus measurement error, further work is required to sufficiently dissect the source of variability contributing to the noted site-based differences identified in the present study. Additionally, multi-site studies must closely monitor and control these potential sources of variability in neuropsychological performance.

We did pursue analyses to determine whether norming procedures for ethnically-divergent youth may have contributed to the site differences. Post hoc testing comparing children who identified as Hispanic/Latino *v.* Non-Hispanic/Latino suggested that differences in ethnic distributions between sites were not the likely cause of our inter-site variability. Of course, it is possible that other demographic differences may have driven the noted site differences; the authors who originally developed the fully normed T-scores did note that certain demographic groups' norms may need further refinement due to an initially small sample (Casaletto et al., 2015). However, we were unable to adequately test for differences between other racially or linguistically-diverse groups in the present study due to the limited demographic diversity of the overall sample. Likewise, our exploratory analyses of age-related differences in reliability were somewhat limited due to small sample sizes per age group, though we did not detect any specific effects of age on reliability. Finally, exploration of sex-related variability in ICCs suggested that males in the present study may have had greater test-retest reliability compared to females in most subtests, though CI were largely overlapping among males and females. Further work is needed to determine the extent to which demographic differences may drive test-retest reliability of the NIH-TB scores.

These findings were largely surprising given earlier reports on the test-retest reliability of NIH-TB measures in children, and the expected robustness of the norming procedures used to derive the T-scores. For instance, Weintraub et al. (2013a, b) reported ICCs ranging from 0.76 to 0.99 in children ages 3–15 years-old (lower bound of 95% CI range: 0.64–0.98); however, these psychometrics were based on raw or computed scores for each subtest, rather than standardized or fully normed scores, and the retest date was between 7 and 21 days after the first test (i.e. very short term). A more recent study assessed long-term stability (average 15.03 ± 3.11 months between retest) of NIH-TB uncorrected standardized scores in older adults (Scott et al., 2019), with results indicating a pattern more similar to our investigation. Namely, reliability indices were predominantly moderate in magnitude, though values ranged from 0.46 to 0.87 for individual tests (lower bound of 95% CI range: 0.14–0.77; Scott et al., 2019). It is possible that the long retest interval of the present study, as well as that of Scott et al., may have attenuated reliability estimates, and that in agreement with previous literature a shorter retest interval may have yielded better stability estimates. Further work is needed to determine the degree to which NIH-TB scores are temporally stable over different time intervals. That said, for many applications (e.g. clinical assessments, longitudinal studies) reliability over longer time intervals is vitally important.

The data in the present study suggest that the fully normed T-scores from the NIH-TB Cognitive Battery may not be suitable for neuropsychological assessments in children and adolescents, especially in the context of long-term, repeated measurements. For instance, a researcher conducting a longitudinal investigation of neurocognitive development may examine composite T-scores across years to determine which children develop improvements or declines in cognitive function relative to their peers. However, a significant change in T-scores may simply be the result of poor reliability of the measure rather than true neurocognitive decline. Such a concern is critical for large-scale studies like ABCD, which intend to track cognitive function and the emergence and progression of mental health disorders throughout adolescence (Luciana et al., 2018). Similar issues arise when considering efficacy studies in clinical trials research, educational programming success, and etcetera. Researchers and clinicians alike must interpret scores with caution, as any long-term shifts in T-scores may be the result of measurement error rather than a clinically-relevant shift in functioning.

Of course, poor reliability also raises questions about each test administration in isolation. The reliability of any neuropsychological assessment is paramount to establishing the *validity* of the instrument; one cannot determine what cognitive construct a test is tapping into without the foundation of consistent measurements (Aldridge et al., 2017; Drost, 2011; Harvey, 2012; Streiner et al., 2015). Thus, the results from the present study raise serious concerns regarding the validity of the NIH-TB Cognitive Battery as an assessment of attention, executive functions, memory, and language abilities over time. Given our findings, researchers and clinicians who work with children are urged to interpret fully normed T-scores and their functional significance with caution. Similar caution is recommended when interpreting the uncorrected and age-corrected standardized scores.

The present study is not without limitations. First, we focused on children and adolescents and did not have a normative adult sample for comparison. Thus, it is difficult to ascertain to what extent our findings are specific to youth. It is possible that, at the two data collection sites of the present study, long-term temporal stability may be excellent for typical adults. Such a finding would suggest that the NIH-TB needs refinement only for administration to children and adolescents, although note that adult studies have raised points of concern as well (e.g. Scott et al., 2019). Second, we did not administer accompanying gold-standard neuropsychological assessments for comparison in the present study. To better interpret the within-person variability over time, it would be helpful to compare test-retest reliability in performance on comparable cognitive tests; one would not expect significant age-related variation in normed scores for gold-standard tests. Third, we were unable to compare reliabilities across linguistically and racially diverse groups given the low diversity in the study sample. Samples were recruited to match the demographic makeup of the surrounding region for each study site (based on census data), which includes predominantly Caucasian, native English-speaking individuals. Further work is needed to decipher demographic differences that may contribute to test-retest reliability. Finally, we did not explore shorter retest periods in the present study. To the best of our knowledge, the fully normed T-scores have yet to be assessed for their short-term test-retest reliability. It is possible (and likely) that shorter-term retest periods may yield stronger stability, thereby supporting the use of fully normed T-scores over limited periods of time. However, the current investigation cannot address this potential strength of the T-scores.

The present study examined the long-term temporal stability of the NIH-TB Cognitive Battery in a large cohort of children and adolescents using fully normed T-scores. Study findings suggested only moderate test-retest reliability over any tested duration, with notable differences in reliability between two data collection sites. Given the unexpectedly low consistency in scores, we recommend further refinement of the NIH-TB Cognitive Battery tool and/or norming procedures before the fully normed T-scores become more widely used as viable resources for determining impairment or tracking longitudinal changes in neurocognitive abilities.

## Supplementary material

For supplementary material accompanying this paper visit https://doi.org/10.1017/S0033291720003487. click here to view supplementary material
