# Supplementary material for: Reliability of the NIH toolbox cognitive battery in children and adolescents: a 3-year longitudinal examination
Source: Psychol Med. 2020 Oct 9;52(9):1718–27. doi: 10.1017/S0033291720003487 (PMC8589010; doi:10.1017/S0033291720003487)
Supplement: Supplementary file 1 [file S0033291720003487sup001.docx]

**Supplemental Results**

**Exploratory Analyses Comparing Ethnicity**

We found multiple site-based differences in test-retest reliability among the NIH-TB Cognitive Battery subtests and composite scores, with site 1 showing stronger consistency than site 2 in each instance. Because site 1 and site 2 significantly differed in the proportion of participants who identified as Hispanic/Latino, we explored whether these ethnic differences in study samples may have driven the noted disparities in reliability between sites. In total, 39 participants identified as Hispanic/Latino across both sites. We identified 39 non-Hispanic Latino participants within our sample who were matched on age (*t* = .144, *p* = .89), sex (χ^2^ = .21, *p* = .65), race (χ^2^ = 3.23, *p* = .52), and mother’s education (χ^2^ = 4.71, *p* = .32), all of which contribute to norming corrections applied to fully normed T-scores. We then calculated ICC absolute agreement measures to determine whether ethnicity may have contributed to the site differences noted in the main text, and in Supplemental Tables 1, 3, and 4. Although there are some tests for which youth who identified as Hispanic/Latino clearly had poorer reliability (e.g., Dimensional Change Card Sorting from year 1 to year 3), there were other tests where the opposite was true (e.g., Flanker from year 2 to year 3); most tests showed similar reliability estimates between ethnic groups with largely overlapping confidence intervals. Importantly, the majority of tests and time periods for which we saw significant site differences showed remarkably similar reliabilities between ethnic groups, suggesting that ethnicity was not likely the driving factor underlying site-based differences in the present study.

**Exploratory Analyses Comparing Age**

To further explore potential demographic characteristics that may have contributed to the generally low stability of NIH-TB subtests, we examined ICC absolute agreement measures within age groups. Specifically, we identified youth who were 9-, 10-, 11-, 12-, 13-, or 14-years-old and older at the start of the study, and separately estimated test-retest reliability within each age group. Youth did not significantly differ by sex (χ^2^ = 3.60, *p* = .61), race (χ^2^ = 45.66, *p* = .11), ethnicity, (χ^2^ = 9.63, *p* = .32), or mother’s education (χ^2^ = 25.51, *p* = .70) across the six specified age groups. Reliability results per age group are reported in Supplemental Table 6. The data indicated widespread variability in test-retest reliability across subtests, time points, and age groups. There was no discernable pattern to suggest that NIH-TB performance metrics may be overall more stable in certain age groups relative to others. That said, the sample size per age group is relatively small (*N*’s range from 20 – 30 for year 1 to year 2, and from 13 – 23 for other periods), thus at least a portion of the instability in our results may be due to inadequate sampling. Further work is needed to determine the extent to which age influences test-retest reliability of the NIH-TB Cognitive Battery.

**Exploratory Analyses Comparing Sex**

In addition to age groupings, we also explored whether sex may have contributed to the variability in reliability metrics over time. Males and females did not significantly differ in age (*t* = .44, *p* = .66), race (χ^2^ = 6.75, *p* = .45), ethnicity, (χ^2^ = .51, *p* = .47), or mother’s education (χ^2^ = 6.16, *p* = .41). Reliability results for males versus females are shown in Supplemental Table 7. Reliability indices were similar between males and females with largely overlapping confidence intervals. However, males did tend to have slightly higher reliability estimates on most NIH-TB subtests when compared to females.

Supplemental Table 1. Reliability metrics by site for the fully-normed T scores of the NIH-TB Cognitive Battery.

|  | ***r*** | | **ICC (3,1) Consistency** | | | | | **ICC (3,1) Absolute Agreement** | | | | | |  |
| --- | --- | --- | --- | --- | --- | --- | --- | --- | --- | --- | --- | --- | --- | --- |
|  |  |  | **ICC** | **Lower Bound** | | **Upper Bound** | | **ICC** | | **Lower Bound** | | **Upper Bound** | |  |
| ***Dimensional Change Card Sort*** | | | | | | | | | | | | | | |
|  | **Year 1 to Year 2** | | | | | | | | | | | | | |
| **All** | .422 | | .421 | .279 | | .545 | | .419 | | .278 | | .543 | |  |
| **Site 1** | .490 | | .490 | .291 | | .648 | | .491 | | .293 | | .649 | |  |
| **Site 2** | .338 | | .337 | .124 | | .521 | | .318 | | .110 | | .502 | |  |
|  | Z = 1.46 | |  |  | |  | |  | |  | |  | |  |
|  | **Year 2 to Year 3** | | | | | | | | | | | | | |
| **All** | .603 | | .597 | .465 | | .704 | | .596 | | .464 | | .702 | |  |
| **Site 1** | .649 | | .638 | .461 | | .766 | | .634 | | .458 | | .763 | |  |
| **Site 2** | .465 | | .464 | .224 | | .651 | | .468 | | .227 | | .654 | |  |
|  | Z = 1.42 | |  |  | |  | |  | |  | |  | |  |
|  | **Year 1 to Year 3** | | | | | | | | | | | | | |
| **All** | .583 | | .580 | .439 | | .693 | | .582 | | .441 | | .694 | |  |
| **Site 1** | .708 | | .704 | .537 | | .818 | | .701 | | .535 | | .815 | |  |
| **Site 2** | .394 | | .394 | .144 | | .597 | | .385 | | .140 | | .587 | |  |
|  | Z = 2.45* | |  |  | |  | |  | |  | |  | |  |
|  |  |  | | |  | |  | |  | |  | |  |  |
| ***Flanker*** | | | | | | | | | | | | | | |
|  | **Year 1 to Year 2** | | | | | | | | | | | | | |
| **All** | .324 | | .322 | .170 | | .459 | | .314 | | .165 | | .450 | |  |
| **Site 1** | .289 | | .289 | .061 | | .488 | | .288 | | .061 | | .486 | |  |
| **Site 2** | .367 | | .362 | .153 | | .540 | | .347 | | .142 | | .525 | |  |
|  | Z = -.69 | |  |  | |  | |  | |  | |  | |  |
|  | **Year 2 to Year 3** | | | | | | | | | | | | | |
| **All** | .421 | | .420 | .256 | | .560 | | .419 | | .256 | | .559 | |  |
| **Site 1** | .437 | | .435 | .207 | | .618 | | .423 | | .198 | | .606 | |  |
| **Site 2** | .419 | | .418 | .169 | | .617 | | .420 | | .171 | | .619 | |  |
|  | Z = .12 | |  |  | |  | |  | |  | |  | |  |
|  | **Year 1 to Year 3** | | | | | | | | | | | | | |
| **All** | .520 | | .516 | .363 | | .642 | | .514 | | .362 | | .640 | |  |
| **Site 1** | .692 | | .691 | .519 | | .809 | | .690 | | .519 | | .808 | |  |
| **Site 2** | .354 | | .343 | .088 | | .556 | | .322 | | .076 | | .535 | |  |
|  | Z = 2.53* | |  |  | |  | |  | |  | |  | |  |
|  |  |  | | |  | |  | |  | |  | |  |  |
| ***List Sorting Working Memory*** | | | | | | | | | | | | | | |
|  | **Year 1 to Year 2** | | | | | | | | | | | | | |
| **All** | .528 | | .527 | .401 | | .634 | | .528 | | .402 | | .635 | |  |
| **Site 1** | .663 | | .658 | .503 | | .772 | | .662 | | .507 | | .775 | |  |
| **Site 2** | .385 | | .385 | .179 | | .559 | | .386 | | .180 | | .560 | |  |
|  | Z = 2.46* | |  |  | |  | |  | |  | |  | |  |
|  | **Year 2 to Year 3** | | | | | | | | | | | | | |
| **All** | .560 | | .555 | .414 | | .670 | | .551 | | .410 | | .667 | |  |
| **Site 1** | .711 | | .697 | .542 | | .807 | | .697 | | .542 | | .806 | |  |
| **Site 2** | .349 | | .348 | .089 | | .564 | | .346 | | .089 | | .561 | |  |
|  | Z = 2.76** | |  |  | |  | |  | |  | |  | |  |
|  | **Year 1 to Year 3** | | | | | | | | | | | | | |
| **All** | .507 | | .494 | .338 | | .625 | | .487 | | .331 | | .618 | |  |
| **Site 1** | .673 | | .651 | .464 | | .782 | | .645 | | .458 | | .778 | |  |
| **Site 2** | .302 | | .297 | .037 | | .520 | | .294 | | .038 | | .515 | |  |
|  | Z = 2.56** | |  |  | |  | |  | |  | |  | |  |
|  |  |  | | |  | |  | |  | |  | |  |  |
|  | ***r*** | | **ICC (3,1) Consistency** | | | | | **ICC (3,1) Absolute Agreement** | | | | | |  |
|  |  |  | **ICC** | **Lower Bound** | | **Upper Bound** | | **ICC** | | **Lower Bound** | | **Upper Bound** | |  |
| ***Pattern Comparison Processing Speed*** | | | | | | | | | | | | | | |
|  | **Year 1 to Year 2** | | | | | | | | | | | | | |
| **All** | .624 | | .623 | .514 | | .712 | | .599 | | .462 | | .703 | |  |
| **Site 1** | .605 | | .605 | .434 | | .734 | | .603 | | .433 | | .732 | |  |
| **Site 2** | .576 | | .576 | .406 | | .707 | | .518 | | .269 | | .688 | |  |
|  | Z = .28 | |  |  | |  | |  | |  | |  | |  |
|  | **Year 2 to Year 3** | | | | | | | | | | | | | |
| **All** | .767 | | .767 | .679 | | .833 | | .755 | | .655 | | .827 | |  |
| **Site 1** | .735 | | .847 | .746 | | .908 | | .735 | | .729 | | .828 | |  |
| **Site 2** | .769 | | .768 | .629 | | .859 | | .856 | | .739 | | .919 | |  |
|  | Z = -.41 | |  |  | |  | |  | |  | |  | |  |
|  | **Year 1 to Year 3** | | | | | | | | | | | | | |
| **All** | .513 | | .509 | .354 | | .636 | | .470 | | .280 | | .619 | |  |
| **Site 1** | .528 | | .528 | .303 | | .697 | | .522 | | .298 | | .692 | |  |
| **Site 2** | .380 | | .378 | .127 | | .583 | | .307 | | .032 | | .535 | |  |
|  | Z = .99 | |  |  | |  | |  | |  | |  | |  |
|  |  |  | | |  | |  | |  | |  | |  |  |
| ***Picture Sequence Memory*** | | | | | | | | | | | | | | |
|  | **Year 1 to Year 2** | | | | | | | | | | | | | |
| **All** | .364 | | .364 | .216 | | .495 | | .340 | | .183 | | .478 | |  |
| **Site 1** | .457 | | .457 | .252 | | .623 | | .449 | | .245 | | .615 | |  |
| **Site 2** | .252 | | .251 | .032 | | .448 | | .220 | | .013 | | .413 | |  |
|  | Z = 1.48 | |  |  | |  | |  | |  | |  | |  |
|  | **Year 2 to Year 3** | | | | | | | | | | | | | |
| **All** | .403 | | .402 | .236 | | .545 | | .402 | | .237 | | .545 | |  |
| **Site 1** | .384 | | .383 | .147 | | .578 | | .383 | | .148 | | .577 | |  |
| **Site 2** | .396 | | .396 | .143 | | .600 | | .400 | | .145 | | .604 | |  |
|  | Z = -.07 | |  |  | |  | |  | |  | |  | |  |
|  | **Year 1 to Year 3** | | | | | | | | | | | | | |
| **All** | .479 | | .477 | .319 | | .611 | | .446 | | .264 | | .594 | |  |
| **Site 1** | .509 | | .504 | .273 | | .680 | | .480 | | .243 | | .663 | |  |
| **Site 2** | .402 | | .402 | .152 | | .603 | | .370 | | .120 | | .577 | |  |
|  | Z = .71 | |  |  | |  | |  | |  | |  | |  |
|  |  |  | | |  | |  | |  | |  | |  |  |
| ***Oral Reading*** | | | | | | | | | | | | | | |
|  | **Year 1 to Year 2** | | | | | | | | | | | | | |
| **All** | .718 | | .718 | .630 | | .788 | | .717 | | .629 | | .787 | |  |
| **Site 1** | .711 | | .713 | .576 | | .811 | | .713 | | .576 | | .811 | |  |
| **Site 2** | .705 | | .705 | .572 | | .802 | | .690 | | .454 | | .794 | |  |
|  | Z = .08 | |  |  | |  | |  | |  | |  | |  |
|  | **Year 2 to Year 3** | | | | | | | | | | | | | |
| **All** | .655 | | .655 | .536 | | .748 | | .646 | | .526 | | .742 | |  |
| **Site 1** | .629 | | .628 | .448 | | .759 | | .627 | | .448 | | .758 | |  |
| **Site 2** | .665 | | .661 | .478 | | .789 | | .642 | | .445 | | .778 | |  |
|  | Z = -.33 | |  |  | |  | |  | |  | |  | |  |
|  | **Year 1 to Year 3** | | | | | | | | | | | | | |
| **All** | .646 | | .638 | .509 | | .728 | | .637 | | .509 | | .737 | |  |
| **Site 1** | .683 | | .670 | .489 | | .795 | | .669 | | .490 | | .794 | |  |
| **Site 2** | .586 | | .586 | .377 | | .738 | | .555 | | .324 | | .721 | |  |
|  | Z = .86 | |  |  | |  | |  | |  | |  | |  |
|  |  |  | | |  | |  | |  | |  | |  |  |
|  | ***r*** | | **ICC (3,1) Consistency** | | | | | **ICC (3,1) Absolute Agreement** | | | | | |  |
|  |  |  | **ICC** | **Lower Bound** | | **Upper Bound** | | **ICC** | | **Lower Bound** | | **Upper Bound** | |  |
| ***Picture Vocabulary*** | | | | | | | | | | | | | | |
|  | **Year 1 to Year 2** | | | | | | | | | | | | | |
| **All** | .608 | | .607 | .495 | | .699 | | .606 | | .494 | | .699 | |  |
| **Site 1** | .642 | | .633 | .470 | | .754 | | .629 | | .466 | | .751 | |  |
| **Site 2** | .588 | | .588 | .421 | | .716 | | .590 | | .424 | | .718 | |  |
|  | Z = .54 | |  |  | |  | |  | |  | |  | |  |
|  | **Year 2 to Year 3** | | | | | | | | | | | | | |
| **All** | .636 | | .528 | .503 | | .728 | | .630 | | .505 | | .729 | |  |
| **Site 1** | .722 | | .714 | .565 | | .818 | | .718 | | .569 | | .821 | |  |
| **Site 2** | .524 | | .517 | .289 | | .689 | | .520 | | .292 | | .692 | |  |
|  | Z = 1.74 | |  |  | |  | |  | |  | |  | |  |
|  | **Year 1 to Year 3** | | | | | | | | | | | | | |
| **All** | .558 | | .556 | .410 | | .674 | | .553 | | .408 | | .671 | |  |
| **Site 1** | .729 | | .729 | .573 | | .834 | | .721 | | .561 | | .829 | |  |
| **Site 2** | .397 | | .389 | .140 | | .592 | | .563 | | .249 | | .746 | |  |
|  | Z = 2.66** | |  |  | |  | |  | |  | |  | |  |
|  |  |  | | |  | |  | |  | |  | |  |  |
| ***Crystallized Composite Score*** | | | | | | | | | | | | | | |
|  | **Year 1 to Year 2** | | | | | | | | | | | | | |
| **All** | .744 | | .744 | .663 | | .808 | | .744 | | .663 | | .808 | |  |
| **Site 1** | .801 | | .794 | .688 | | .866 | | .795 | | .691 | | .867 | |  |
| **Site 2** | .700 | | .693 | .557 | | .793 | | .693 | | .558 | | .792 | |  |
|  | Z = 1.47 | |  |  | |  | |  | |  | |  | |  |
|  | **Year 2 to Year 3** | | | | | | | | | | | | | |
| **All** | .747 | | .746 | .653 | | .817 | | .744 | | .650 | | .815 | |  |
| **Site 1** | .758 | | .758 | .626 | | .847 | | .758 | | .628 | | .847 | |  |
| **Site 2** | .730 | | .728 | .577 | | .831 | | .725 | | .573 | | .829 | |  |
|  | Z = .33 | |  |  | |  | |  | |  | |  | |  |
|  | **Year 1 to Year 3** | | | | | | | | | | | | | |
| **All** | .665 | | .663 | .544 | | .756 | | .660 | | .540 | | .753 | |  |
| **Site 1** | .829 | | .828 | .720 | | .897 | | .830 | | .723 | | .898 | |  |
| **Site 2** | .520 | | .511 | .290 | | .679 | | .504 | | .285 | | .673 | |  |
|  | Z = 3.20** | |  |  | |  | |  | |  | |  | |  |
|  |  |  | | |  | |  | |  | |  | |  |  |
| ***Fluid Composite Score*** | | | | | | | | | | | | | | |
|  | **Year 1 to Year 2** | | | | | | | | | | | | | |
| **All** | .591 | | .590 | .474 | | .685 | | .585 | | .469 | | .682 | |  |
| **Site 1** | .730 | | .726 | .595 | | .820 | | .726 | | .595 | | .820 | |  |
| **Site 2** | .392 | | .392 | .187 | | .564 | | .387 | | .185 | | .559 | |  |
|  | Z = 3.22** | |  |  | |  | |  | |  | |  | |  |
|  | **Year 2 to Year 3** | | | | | | | | | | | | | |
| **All** | .716 | | .716 | .614 | | .794 | | .703 | | .591 | | .787 | |  |
| **Site 1** | .712 | | .711 | .561 | | .816 | | .692 | | .522 | | .806 | |  |
| **Site 2** | .686 | | .686 | .517 | | .803 | | .682 | | .514 | | .800 | |  |
|  | Z = .27 | |  |  | |  | |  | |  | |  | |  |
|  | **Year 1 to Year 3** | | | | | | | | | | | | | |
| **All** | .612 | | .612 | .480 | | .717 | | .590 | | .440 | | .706 | |  |
| **Site 1** | .751 | | .748 | .600 | | .846 | | .713 | | .505 | | .835 | |  |
| **Site 2** | .389 | | .387 | .142 | | .587 | | .379 | | .139 | | .578 | |  |
|  | Z = 2.97** | |  |  | |  | |  | |  | |  | |  |
|  |  |  | | |  | |  | |  | |  | |  |  |
|  | ***r*** | | **ICC (3,1) Consistency** | | | | | **ICC (3,1) Absolute Agreement** | | | | | |  |
|  |  |  | **ICC** | **Lower Bound** | | **Upper Bound** | | **ICC** | | **Lower Bound** | | **Upper Bound** | |  |
| ***Total Composite Score*** | | | | | | | | | | | | | | |
|  | **Year 1 to Year 2** | | | | | | | | | | | | | |
| **All** | .722 | | .722 | .635 | | .790 | | .717 | | .629 | | .787 | |  |
| **Site 1** | .836 | | .828 | .738 | | .889 | | .827 | | .737 | | .889 | |  |
| **Site 2** | .600 | | .590 | .424 | | .717 | | .585 | | .419 | | .713 | |  |
|  | Z = 3.22** | |  |  | |  | |  | |  | |  | |  |
|  | **Year 2 to Year 3** | | | | | | | | | | | | | |
| **All** | .772 | | .772 | .687 | | .836 | | .759 | | .660 | | .830 | |  |
| **Site 1** | .782 | | .780 | .659 | | .862 | | .768 | | .633 | | .856 | |  |
| **Site 2** | .735 | | .733 | .584 | | .834 | | .723 | | .586 | | .828 | |  |
|  | Z = .58 | |  |  | |  | |  | |  | |  | |  |
|  | **Year 2 to Year 3** | | | | | | | | | | | | | |
| **All** | .626 | | .624 | .495 | | .726 | | .608 | | .468 | | .717 | |  |
| **Site 1** | .811 | | .804 | .684 | | .882 | | .788 | | .644 | | .875 | |  |
| **Site 2** | .414 | | .395 | .151 | | .593 | | .384 | | .146 | | .582 | |  |
|  | Z = 3.63*** | |  |  | |  | |  | |  | |  | |  |

Statistical significance was only calculated for *Z* score transformations comparing Pearson correlations between sites: **p* < .05; ***p* < .01, ****p* < .001

*N*’s for Year 1 to Year 2: *N*_all_ = 162, *N*_site1_ = 83, *N*_site 2_ = 79; *N*’s for Year 2 to Year 3: *N*_all_ = 118, *N*_site1_ = 65, *N*_site 2_ = 53; *N*’s for Year 1 to Year 3: *N*_all_ = 118, *N*_site1_ = 65, *N*_site 2_ = 53

Supplemental Table 2. Number of participants whose fully normed T scores deviated by at least +/- 1 standard deviation (SD) across years of the study for each subtest and composite score of the NIH-TB Cognitive Battery.

|  | **Year 1 to Year 2** | | | |  | **Year 2 to Year 3** | | | |  | **Year 1 to Year 3** | | | |
| --- | --- | --- | --- | --- | --- | --- | --- | --- | --- | --- | --- | --- | --- | --- |
|  | **+1 SD** | **-1 SD** | **Total N** | **% of**  **Sample** |  | **+1 SD** | **-1 SD** | **Total**  **N** | **% of**  **Sample** |  | **+1 SD** | **-1 SD** | **Total**  **N** | **% of**  **Sample** |
| **DCCS** | 29 | 21 | 50 | 30.67% |  | 13 | 24 | 37 | 31.36% |  | 32 | 22 | 54 | 45.76% |
| **Flanker** | 25 | 12 | 37 | 22.70% |  | 10 | 13 | 23 | 19.49% |  | 25 | 9 | 34 | 28.81% |
| **List WM** | 21 | 26 | 47 | 28.83% |  | 7 | 19 | 26 | 22.03% |  | 37 | 16 | 53 | 44.92% |
| **Proc Speed** | 16 | 48 | 64 | 39.26% |  | 6 | 20 | 26 | 22.03% |  | 52 | 40 | 92 | 77.97% |
| **Pic Mem** | 16 | 51 | 67 | 41.10% |  | 21 | 30 | 51 | 43.22% |  | 50 | 30 | 80 | 67.80% |
| **Oral Read** | 11 | 13 | 24 | 14.72% |  | 9 | 14 | 23 | 19.49% |  | 33 | 16 | 49 | 41.53% |
| **Pic Vocab** | 20 | 26 | 46 | 28.22% |  | 18 | 12 | 30 | 25.42% |  | 37 | 17 | 54 | 45.76% |
| **Crystal** | 7 | 19 | 26 | 15.95% |  | 9 | 14 | 23 | 19.49% |  | 35 | 12 | 47 | 39.83% |
| **Fluid** | 13 | 27 | 40 | 24.54% |  | 10 | 17 | 27 | 22.88% |  | 38 | 24 | 62 | 52.54% |
| **Total** | 6 | 18 | 24 | 14.72% |  | 6 | 14 | 20 | 16.95% |  | 45 | 24 | 69 | 58.47% |

Note: +1 SD indicates the number of participants whose fully normed T score increased by at least 1 standard deviation (10 points) in a subsequent year; -1 SD indicates the number of participants whose fully normed T score decreased by at least 1 standard deviation (10 points) in a subsequent year; Total N indicates the total number of participants whose fully normed T score increased OR decreased by at least 1 standard deviation (10 points) across years; % of sample indicates the percentage of the total sample with data across the two indicated years whose fully normed T scores deviated by at least 1 standard deviation.

“DCCS” = dimensional change card sorting; “List WM” = list sorting working memory; “Proc Speed” = pattern comparison processing speed; “Pic Mem” = picture sequence memory; “Oral Read” = oral reading; “Pic Vocab” = picture vocabulary; “Crystal” = crystallized cognition composite; “Fluid” = fluid cognition composite; “Total” = total cognition composite

Supplemental Table 3. Reliability metrics by site for the NIH-TB Cognitive Battery’s uncorrected standard scores

|  | ***r*** | **ICC (3,1) Consistency** | | | | | | **ICC (3,1) Absolute Agreement** | | | | | | |  |
| --- | --- | --- | --- | --- | --- | --- | --- | --- | --- | --- | --- | --- | --- | --- | --- |
|  |  | **ICC** | | **Lower Bound** | | **Upper Bound** | | **ICC** | | **Lower Bound** | | **Upper Bound** | | |  |
| ***Dimensional Change Card Sort*** | | | | | | | | | | | | | | | |
|  | **Year 1 to Year 2** | | | | | | | | | | | | | | |
| **All** | .457 | .454 | | .323 | | .568 | | .451 | | .320 | | .564 | | |  |
| **Site 1** | .538 | .536 | | .363 | | .673 | | .512 | | .327 | | .658 | | |  |
| **Site 2** | .380 | .375 | | .171 | | .549 | | .378 | | .172 | | .552 | | |  |
|  | Z = 1.26 |  | |  | |  | |  | |  | |  | | |  |
|  | **Year 2 to Year 3** | | | | | | | | | | | | | | |
| **All** | .595 | .595 | | .464 | | .700 | | .568 | | .411 | | .688 | | |  |
| **Site 1** | .568 | .568 | | .377 | | .712 | | .539 | | .329 | | .695 | | |  |
| **Site 2** | .550 | .550 | | .330 | | .713 | | .528 | | .301 | | .698 | | |  |
|  | Z = .14 | |  | |  | |  | |  | |  | |  |  |  |
|  | **Year 1 to Year 3** | | | | | | | | | | | | | | |
| **All** | .534 | .528 | | .386 | | .645 | | .484 | | .292 | | .630 | | |  |
| **Site 1** | .596 | .591 | | .406 | | .729 | | .511 | | .198 | | .706 | | |  |
| **Site 2** | .445 | .433 | | .194 | | .623 | | .417 | | .181 | | .609 | | |  |
|  | Z = 1.10 | |  | |  | |  | |  | |  | |  |  |  |
| ***Flanker*** | | | | | | | | | | | | | | | |
|  | **Year 1 to Year 2** | | | | | | | | | | | | | | |
| **All** | .421 | .416 | | .280 | | .535 | | .417 | | .281 | | .536 | | |  |
| **Site 1** | .367 | .361 | | .158 | | .534 | | .363 | | .160 | | .536 | | |  |
| **Site 2** | .456 | .451 | | .258 | | .610 | | .454 | | .260 | | .612 | | |  |
|  | Z = -.67 |  | |  | |  | |  | |  | |  | | |  |
|  | **Year 2 to Year 3** | | | | | | | | | | | | | | |
| **All** | .475 | .475 | | .322 | | .603 | | .450 | | .284 | | .588 | | |  |
| **Site 1** | .418 | .418 | | .195 | | .599 | | .362 | | .109 | | .565 | | |  |
| **Site 2** | .485 | .483 | | .247 | | .665 | | .483 | | .248 | | .798 | | |  |
|  | Z = -.44 | |  | |  | |  | |  | |  | |  |  |  |
|  | **Year 1 to Year 3** | | | | | | | | | | | | | | |
| **All** | .461 | .449 | | .294 | | .580 | | .415 | | .237 | | .562 | | |  |
| **Site 1** | .486 | .468 | | .255 | | .638 | | .405 | | .138 | | .607 | | |  |
| **Site 2** | .404 | .391 | | .145 | | .592 | | .380 | | .139 | | .580 | | |  |
|  | Z = .54 | |  | |  | |  | |  | |  | |  |  |  |
| ***List Sorting Working Memory*** | | | | | | | | | | | | | | | |
|  | **Year 1 to Year 2** | | | | | | | | | | | | | | |
| **All** | .627 | .626 | | .523 | | .711 | | .608 | | .487 | | .703 | | |  |
| **Site 1** | .663 | .663 | | .523 | | .769 | | .654 | | .508 | | .762 | | |  |
| **Site 2** | .586 | .586 | | .422 | | .713 | | .559 | | .373 | | .699 | | |  |
|  | Z = .79 |  | |  | |  | |  | |  | |  | | |  |
|  | **Year 2 to Year 3** | | | | | | | | | | | | | | |
| **All** | .627 | .625 | | .501 | | .723 | | .601 | | .454 | | .713 | | |  |
| **Site 1** | .712 | .706 | | .559 | | .810 | | .694 | | .539 | | .802 | | |  |
| **Site 2** | .514 | .514 | | .285 | | .687 | | .481 | | .237 | | .666 | | |  |
|  | Z = 1.70 | |  | |  | |  | |  | |  | |  |  |  |
|  | **Year 1 to Year 3** | | | | | | | | | | | | | | |
| **All** | .518 | .514 | | .370 | | .634 | | .443 | | .195 | | .619 | | |  |
| **Site 1** | .596 | .589 | | .405 | | .728 | | .528 | | .258 | | .707 | | |  |
| **Site 2** | .422 | .421 | | .179 | | .614 | | .348 | | .065 | | .572 | | |  |
|  | Z = 1.25 | |  | |  | |  | |  | |  | |  |  |  |
|  | | | | | | | | | | | | | | | |
|  | ***r*** | **ICC (3,1) Consistency** | | | | | | **ICC (3,1) Absolute Agreement** | | | | | | |  |
|  |  | **ICC** | | **Lower Bound** | | **Upper Bound** | | **ICC** | | **Lower Bound** | | **Upper Bound** | | |  |
| ***Processing Speed*** | | | | | | | | | | | | | | | |
|  | **Year 1 to Year 2** | | | | | | | | | | | | | | |
| **All** | .704 | .617 | | .774 | | .704 | | .642 | | .400 | | .776 | | |  |
| **Site 1** | .688 | .687 | | .554 | | .880 | | .661 | | .495 | | .775 | | |  |
| **Site 2** | .686 | .684 | | .547 | | .785 | | .569 | | .148 | | .772 | | |  |
|  | Z = .02 |  | |  | |  | |  | |  | |  | | |  |
|  | **Year 2 to Year 3** | | | | | | | | | | | | | | |
| **All** | .810 | .810 | | .738 | | .864 | | .757 | | .513 | | .865 | | |  |
| **Site 1** | .771 | .771 | | .650 | | .854 | | .715 | | .447 | | .845 | | |  |
| **Site 2** | .819 | .819 | | .706 | | .891 | | .765 | | .484 | | .883 | | |  |
|  | Z = -.69 |  | |  | |  | |  | |  | |  | | |  |
|  | **Year 1 to Year 3** | | | | | | | | | | | | | | |
| **All** | .663 | .631 | | .510 | | .727 | | .490 | | .058 | | .718 | | |  |
| **Site 1** | .614 | .612 | | .434 | | .744 | | .514 | | .157 | | .722 | | |  |
| **Site 2** | .573 | .572 | | .366 | | .725 | | .373 | | -070 | | .798 | | |  |
|  | Z = .33 |  | |  | |  | |  | |  | |  | | |  |
| ***Picture Sequence Memory*** | | | | | | | | | | | | | | | |
|  | **Year 1 to Year 2** | | | | | | | | | | | | | | |
| **All** | .430 | .430 | | .296 | | .547 | | .393 | | .227 | | .531 | | |  |
| **Site 1** | .488 | .488 | | .305 | | .636 | | .472 | | .287 | | .623 | | |  |
| **Site 2** | .354 | .354 | | .147 | | .531 | | .299 | | .069 | | .494 | | |  |
|  | Z = 1.02 |  | |  | |  | |  | |  | |  | | |  |
|  | **Year 2 to Year 3** | | | | | | | | | | | | | | |
| **All** | .452 | .451 | | .295 | | .584 | | .448 | | .292 | | .580 | | |  |
| **Site 1** | .465 | .465 | | .252 | | .636 | | .460 | | .248 | | .360 | | |  |
| **Site 2** | .399 | .396 | | .143 | | .600 | | .398 | | .145 | | .602 | | |  |
|  | Z = .43 |  | |  | |  | |  | |  | |  | | |  |
|  | **Year 1 to Year 3** | | | | | | | | | | | | | | |
| **All** | .530 | .529 | | .387 | | .647 | | .462 | | .217 | | .633 | | |  |
| **Site 1** | .477 | .475 | | .263 | | .643 | | .427 | | .183 | | .616 | | |  |
| **Site 2** | .532 | .531 | | .311 | | .697 | | .441 | | .112 | | .663 | | |  |
|  | Z = -.39 |  | |  | |  | |  | |  | |  | | |  |
| ***Oral Reading*** | | | | | | | | | | | | | | | |
|  | **Year 1 to Year 2** | | | | | | | | | | | | | | |
| **All** | .809 | .808 | | .747 | | .855 | | .774 | | .627 | | .856 | | |  |
| **Site 1** | .739 | .736 | | .619 | | .821 | | .729 | | .609 | | .816 | | |  |
| **Site 2** | .866 | .865 | | .797 | | .911 | | .789 | | .357 | | .909 | | |  |
|  | Z = -2.31^*^ |  | |  | |  | |  | |  | |  | | |  |
|  | **Year 2 to Year 3** | | | | | | | | | | | | | | |
| **All** | .734 | .734 | | .638 | | .807 | | .677 | | .439 | | .805 | | |  |
| **Site 1** | .651 | .650 | | .484 | | .771 | | .593 | | .330 | | .754 | | |  |
| **Site 2** | .769 | .768 | | .630 | | .859 | | .712 | | .431 | | .848 | | |  |
|  | Z = -1.27 |  | |  | |  | |  | |  | |  | | |  |
|  | **Year 1 to Year 3** | | | | | | | | | | | | | | |
| **All** | .748 | .742 | | .649 | | .813 | | .645 | | .255 | | .813 | |  |  |
| **Site 1** | .663 | .655 | | .490 | | .774 | | .601 | | .347 | | .758 | |  |  |
| **Site 2** | .788 | .788 | | .662 | | .871 | | .620 | | -.007 | | .845 | |  |  |
|  | Z = -1.41 |  | |  | |  | |  | |  | |  | | |  |
|  | ***r*** | **ICC (3,1) Consistency** | | | | | | **ICC (3,1) Absolute Agreement** | | | | | | |  |
|  |  | **ICC** | | **Lower Bound** | | **Upper Bound** | | **ICC** | | **Lower Bound** | | **Upper Bound** | | |  |
| ***Picture Vocabulary*** | | | | | | | | | | | | | | | |
|  | **Year 1 to Year 2** | | | | | | | | | | | | | | |
| **All** | .735 | .734 | | .655 | | .798 | | .699 | | .546 | | .795 | | |  |
| **Site 1** | .714 | .713 | | .588 | | .804 | | .681 | | .507 | | .794 | | |  |
| **Site 2** | .742 | .742 | | .624 | | .826 | | .704 | | .513 | | .817 | | |  |
|  | Z = -.37 |  | |  | |  | |  | |  | |  | | |  |
|  | **Year 2 to Year 3** | | | | | | | | | | | | | | |
| **All** | .765 | .764 | | .677 | | .830 | | .735 | | .593 | | .825 | | |  |
| **Site 1** | .761 | .758 | | .632 | | .845 | | .720 | | .515 | | .836 | | |  |
| **Site 2** | .762 | .762 | | .620 | | .855 | | .743 | | .578 | | .847 | | |  |
|  | Z = -.01 |  | |  | |  | |  | |  | |  | | |  |
|  | **Year 1 to Year 3** | | | | | | | | | | | | | | |
| **All** | .737 | .734 | | .640 | | .807 | | .603 | | .115 | | .804 | | |  |
| **Site 1** | .700 | .690 | | .538 | | .799 | | .576 | | .144 | | .782 | | |  |
| **Site 2** | .759 | .758 | | .620 | | .851 | | .607 | | .037 | | .828 | | |  |
|  | Z = -.67 |  | |  | |  | |  | |  | |  | | |  |
| ***Crystalized Composite*** | | | | | | | | | | | | | | | |
|  | **Year 1 to Year 2** | | | | | | | | | | | | | | |
| **All** | .858 | .855 | | .807 | | .891 | | .813 | | .615 | | .895 | | |  |
| **Site 1** | .825 | .820 | | .735 | | .880 | | .796 | | .660 | | .875 | | |  |
| **Site 2** | .878 | .877 | | .814 | | .919 | | .815 | | .457 | | .917 | | |  |
|  | Z = -1.22 |  | |  | |  | |  | |  | |  | | |  |
|  | **Year 2 to Year 3** | | | | | | | | | | | | | | |
| **All** | .837 | .837 | | .774 | | .883 | | .777 | | .484 | | .886 | | |  |
| **Site 1** | .794 | .794 | | .684 | | .869 | | .727 | | .405 | | .862 | | |  |
| **Site 2** | .856 | .856 | | .766 | | .913 | | .800 | | .492 | | .907 | | |  |
|  | Z = -1.03 |  | |  | |  | |  | |  | |  | | |  |
|  | **Year 1 to Year 3** | | | | | | | | | | | | | | |
| **All** | .855 | .850 | | .792 | | .892 | | .692 | | .002 | | .882 | | |  |
| **Site 1** | .883 | .824 | | .727 | | .889 | | .699 | | .110 | | .877 | | |  |
| **Site 2** | .872 | .872 | | .792 | | .922 | | .663 | | -.075 | | .886 | | |  |
|  | Z = .25 |  | |  | |  | |  | |  | |  | | |  |
| ***Fluid Composite*** | | | | | | | | | | | | | | | |
|  | **Year 1 to Year 2** | | | | | | | | | | | | | | |
| **All** | .772 | .772 | | .701 | | .827 | | .701 | | .403 | | .833 | | |  |
| **Site 1** | .779 | .779 | | .678 | | .852 | | .733 | | .513 | | .846 | | |  |
| **Site 2** | .742 | .740 | | .621 | | .825 | | .636 | | .212 | | .816 | | |  |
|  | Z = .55 |  | |  | |  | |  | |  | |  | | |  |
|  | **Year 2 to Year 3** | | | | | | | | | | | | | | |
| **All** | .784 | .784 | | .704 | | .844 | | .719 | | .436 | | .845 | | |  |
| **Site 1** | .762 | .761 | | .636 | | .847 | | .683 | | .328 | | .837 | | |  |
| **Site 2** | .768 | .768 | | .634 | | .857 | | .714 | | .446 | | .846 | | |  |
|  | Z = -.08 |  | |  | |  | |  | |  | |  | | |  |
|  | **Year 1 to Year 3** | | | | | | | | | | | | | | |
| **All** | .746 | .745 | | .655 | | .814 | | .536 | | -.058 | | .793 | | |  |
| **Site 1** | .734 | .731 | | .594 | | .827 | | .532 | | -.051 | | .793 | | |  |
| **Site 2** | .697 | .697 | | .536 | | .809 | | .463 | | -.085 | | .755 | | |  |
|  | Z = .40 |  | |  | |  | |  | |  | |  | | |  |
|  | ***r*** | **ICC (3,1) Consistency** | | | | | | **ICC (3,1) Absolute Agreement** | | | | | | |  |
|  |  | **ICC** | | **Lower Bound** | | **Upper Bound** | | **ICC** | | **Lower Bound** | | **Upper Bound** | | |  |
| ***Total Composite Score*** | | | | | | | | | | | | | | | |
|  | **Year 1 to Year 2** | | | | | | | | | | | | | | |
| **All** | .869 | .867 | | .823 | | .901 | | .789 | | .359 | | .906 | | |  |
| **Site 1** | .837 | .835 | | .757 | | .890 | | .785 | | .531 | | .888 | | |  |
| **Site 2** | .790 | .890 | | .833 | | .928 | | .772 | | .084 | | .918 | | |  |
|  | Z = .87 |  | |  | |  | |  | |  | |  | | |  |
|  | **Year 2 to Year 3** | | | | | | | | | | | | | | |
| **All** | .857 | .856 | | .800 | | .898 | | .768 | | .297 | | .899 | | |  |
| **Site 1** | .820 | .819 | | .719 | | .886 | | .717 | | .217 | | .876 | | |  |
| **Site 2** | .867 | .865 | | .781 | | .919 | | .784 | | .311 | | .911 | | |  |
|  | Z = -.86 |  | |  | |  | |  | |  | |  | | |  |
|  | **Year 1 to Year 3** | | | | | | | | | | | | | | |
| **All** | .835 | .826 | | .760 | | .875 | | .589 | | -.083 | | .843 | | |  |
| **Site 1** | .795 | .785 | | .670 | | .863 | | .571 | | -.069 | | .827 | | |  |
| **Site 2** | .838 | .835 | | .736 | | .899 | | .543 | | -.090 | | .833 | | |  |
|  | Z = -.68 |  | |  | |  | |  | |  | |  | | |  |

Statistical significance was only calculated for *Z* score transformations comparing Pearson correlations between sites: **p* < .05; ***p* < .01, ****p* < .001

*N*’s for Year 1 to Year 2: *N*_all_ = 163, *N*_site1_ = 83, *N*_site 2_ = 80; *N*’s for Year 2 to Year 3: *N*_all_ = 118, *N*_site1_ = 65, *N*_site 2_ = 53; *N*’s for Year 1 to Year 3: *N*_all_ = 118, *N*_site1_ = 65, *N*_site 2_ = 53

Supplemental Table 4. Reliability metrics by site for the NIH-TB Cognitive Battery’s age-corrected standard scores

|  | ***r*** | **ICC (3,1) Consistency** | | | **ICC (3,1) Absolute Agreement** | | |  |
| --- | --- | --- | --- | --- | --- | --- | --- | --- |
|  |  | **ICC** | **Lower Bound** | **Upper Bound** | **ICC** | **Lower Bound** | **Upper Bound** |  |
| ***Dimensional Change Card Sort*** | | | | | | | | |
|  | **Year 1 to Year 2** | | | | | | | |
| **All** | .401 | .401 | .264 | .522 | .400 | .263 | .521 |  |
| **Site 1** | .546 | .546 | .375 | .681 | .543 | .373 | .678 |  |
| **Site 2** | .244 | .244 | .027 | .439 | .228 | .022 | .418 |  |
|  | Z = 2.28^*^ |  |  |  |  |  |  |  |
|  | **Year 2 to Year 3** | | | | | | | |
| **All** | .572 | .567 | .431 | .678 | .566 | .430 | .676 |  |
| **Site 1** | .648 | .634 | .463 | .760 | .628 | .457 | .755 |  |
| **Site 2** | .369 | .369 | .112 | .580 | .373 | .114 | .584 |  |
|  | Z = 2.02^*^ |  |  |  |  |  |  |  |
|  | **Year 1 to Year 3** | | | | | | | |
| **All** | .515 | .512 | .368 | .632 | .514 | .370 | .634 |  |
| **Site 1** | .650 | .646 | .478 | .768 | .663 | .460 | .760 |  |
| **Site 2** | .336 | .336 | .082 | .549 | .323 | .078 | .534 |  |
|  | Z = 2.24^*^ |  |  |  |  |  |  |  |
| ***Flanker*** | | | | | | | | |
|  | **Year 1 to Year 2** | | | | | | | |
| **All** | .374 | .371 | .230 | .496 | .360 | .219 | .486 |  |
| **Site 1** | .343 | .342 | .137 | .518 | .338 | .136 | .514 |  |
| **Site 2** | .407 | .397 | .196 | .567 | .379 | .179 | .550 |  |
|  | Z = -.47 |  |  |  |  |  |  |  |
|  | **Year 2 to Year 3** | | | | | | | |
| **All** | .469 | .468 | .314 | .597 | .466 | .313 | .595 |  |
| **Site 1** | .487 | .484 | .274 | .650 | .468 | .257 | .637 |  |
| **Site 2** | .414 | .413 | .164 | .614 | .417 | .166 | .617 |  |
|  | Z = .48 |  |  |  |  |  |  |  |
|  | **Year 1 to Year 3** | | | | | | | |
| **All** | .527 | .525 | .383 | .643 | .525 | .383 | .643 |  |
| **Site 1** | .671 | .671 | .512 | .786 | .671 | .513 | .785 |  |
| **Site 2** | .364 | .351 | .100 | .561 | .338 | .094 | .546 |  |
|  | Z = 2.27^*^ |  |  |  |  |  |  |  |
| ***List Sorting Working Memory*** | | | | | | | | |
|  | **Year 1 to Year 2** | | | | | | | |
| **All** | .519 | .518 | .396 | .622 | .519 | .397 | .623 |  |
| **Site 1** | .658 | .658 | .517 | .765 | .660 | .519 | .766 |  |
| **Site 2** | .362 | .359 | .152 | .535 | .361 | .153 | .538 |  |
|  | Z = 1.23 |  |  |  |  |  |  |  |
|  | **Year 2 to Year 3** | | | | | | | |
| **All** | .599 | .597 | .467 | .702 | .593 | .462 | .698 |  |
| **Site 1** | .701 | .690 | .538 | .799 | .692 | .541 | .800 |  |
| **Site 2** | .452 | .450 | .207 | .641 | .440 | .201 | .631 |  |
|  | Z = 2.01^*^ |  |  |  |  |  |  |  |
|  | **Year 1 to Year 3** | | | | | | | |
| **All** | .495 | .484 | .335 | .609 | .478 | .329 | .604 |  |
| **Site 1** | .621 | .609 | .430 | .741 | .660 | .420 | .735 |  |
| **Site 2** | .349 | .340 | .087 | .552 | .340 | .088 | .551 |  |
|  | Z = 1.91 |  |  |  |  |  |  |  |
|  | | | | | | | | |
|  | ***r*** | **ICC (3,1) Consistency** | | | **ICC (3,1) Absolute Agreement** | | |  |
|  |  | **ICC** | **Lower Bound** | **Upper Bound** | **ICC** | **Lower Bound** | **Upper Bound** |  |
| ***Processing Speed*** | | | | | | | | |
|  | **Year 1 to Year 2** | | | | | | | |
| **All** | .643 | .643 | .543 | .725 | .617 | .483 | .717 |  |
| **Site 1** | .624 | .623 | .471 | .739 | .616 | .46 | .734 |  |
| **Site 2** | .587 | .587 | .423 | .714 | .532 | .291 | .696 |  |
|  | Z = .37 |  |  |  |  |  |  |  |
|  | **Year 2 to Year 3** | | | | | | | |
| **All** | .770 | .770 | .684 | .834 | .756 | .655 | .828 |  |
| **Site 1** | .734 | .734 | .598 | .829 | .721 | .575 | .822 |  |
| **Site 2** | .762 | .760 | .618 | .854 | .747 | .592 | .847 |  |
|  | Z = -.33 |  |  |  |  |  |  |  |
|  | **Year 1 to Year 3** | | | | | | | |
| **All** | .574 | .572 | .439 | .681 | .520 | .309 | .669 |  |
| **Site 1** | .554 | .554 | .359 | .702 | .527 | .318 | .685 |  |
| **Site 2** | .470 | .469 | .237 | .650 | .386 | .083 | .611 |  |
|  | Z = .60 |  |  |  |  |  |  |  |
| ***Picture Sequence Memory*** | | | | | | | | |
|  | **Year 1 to Year 2** | | | | | | | |
| **All** | .412 | .415 | .276 | .531 | .390 | .244 | .517 |  |
| **Site 1** | .454 | .453 | .264 | .608 | .443 | .255 | .599 |  |
| **Site 2** | .337 | .336 | .127 | .517 | .305 | .096 | .490 |  |
|  | Z = .87 |  |  |  |  |  |  |  |
|  | **Year 2 to Year 3** | | | | | | | |
| **All** | .425 | .424 | .264 | .562 | .423 | .264 | .560 |  |
| **Site 1** | .410 | .410 | .186 | .593 | .406 | .185 | .589 |  |
| **Site 2** | .395 | .394 | .141 | .599 | .398 | .143 | .603 |  |
|  | Z = .09 |  |  |  |  |  |  |  |
|  | **Year 1 to Year 3** | | | | | | | |
| **All** | .517 | .511 | .366 | .632 | .472 | .286 | .616 |  |
| **Site 1** | .483 | .476 | .264 | .644 | .437 | .204 | .620 |  |
| **Site 2** | .493 | .490 | .260 | .667 | .455 | .211 | .644 |  |
|  | Z = -.07 |  |  |  |  |  |  |  |
| ***Oral Reading*** | | | | | | | | |
|  | **Year 1 to Year 2** | | | | | | | |
| **All** | .745 | .743 | .665 | .805 | .744 | .666 | .805 |  |
| **Site 1** | .757 | .757 | .647 | .836 | .757 | .648 | .836 |  |
| **Site 2** | .699 | .697 | .565 | .795 | .692 | .558 | .791 |  |
|  | Z = .78 |  |  |  |  |  |  |  |
|  | **Year 2 to Year 3** | | | | | | | |
| **All** | .703 | .702 | .567 | .783 | .692 | .580 | .777 |  |
| **Site 1** | .637 | .629 | .456 | .756 | .625 | .453 | .753 |  |
| **Site 2** | .724 | .723 | .564 | .830 | .705 | .529 | .821 |  |
|  | Z = -.86 |  |  |  |  |  |  |  |
|  | **Year 1 to Year 3** | | | | | | | |
| **All** | .625 | .609 | .483 | .710 | .609 | .483 | .710 |  |
| **Site 1** | .629 | .602 | .422 | .737 | .606 | .425 | .740 |  |
| **Site 2** | .546 | .542 | .325 | .705 | .534 | .318 | .698 |  |
|  | Z = .67 |  |  |  |  |  |  |  |
|  | ***r*** | **ICC (3,1) Consistency** | | | **ICC (3,1) Absolute Agreement** | | |  |
|  |  | **ICC** | **Lower Bound** | **Upper Bound** | **ICC** | **Lower Bound** | **Upper Bound** |  |
| ***Picture Vocabulary*** | | | | | | | | |
|  | **Year 1 to Year 2** | | | | | | | |
| **All** | .633 | .631 | .529 | .715 | .632 | .530 | .716 |  |
| **Site 1** | .646 | .630 | .480 | .744 | .628 | .478 | .742 |  |
| **Site 2** | .623 | .622 | .467 | 740 | .625 | .470 | .742 |  |
|  | Z = .24 |  |  |  |  |  |  |  |
|  | **Year 2 to Year 3** | | | | | | | |
| **All** | .684 | .678 | .567 | .764 | .679 | .569 | .766 |  |
| **Site 1** | .709 | .706 | .560 | .810 | .709 | .564 | .812 |  |
| **Site 2** | .641 | .627 | .431 | .766 | .631 | .435 | .769 |  |
|  | Z = .66 |  |  |  |  |  |  |  |
|  | **Year 1 to Year 3** | | | | | | | |
| **All** | .568 | .566 | .432 | .676 | .566 | .432 | .676 |  |
| **Site 1** | .658 | .654 | .489 | .774 | .649 | .484 | .770 |  |
| **Site 2** | .497 | .479 | .249 | .657 | .483 | .252 | .661 |  |
|  | Z = 1.28 |  |  |  |  |  |  |  |
| ***Crystalized Composite*** | | | | | | | | |
|  | **Year 1 to Year 2** | | | | | | | |
| **All** | .793 | .793 | 728 | .844 | .794 | .729 | .844 |  |
| **Site 1** | .847 | .843 | .767 | .895 | .844 | .769 | .897 |  |
| **Site 2** | .738 | .732 | .611 | .819 | .73 | .613 | .820 |  |
|  | Z = 1.88 |  |  |  |  |  |  |  |
|  | **Year 2 to Year 3** | | | | | | | |
| **All** | .813 | .810 | .739 | .864 | .807 | .733 | .861 |  |
| **Site 1** | .776 | .773 | .653 | .855 | .773 | .654 | .855 |  |
| **Site 2** | .826 | .823 | .716 | .892 | .817 | .704 | .889 |  |
|  | Z = -.74 |  |  |  |  |  |  |  |
|  | **Year 1 to Year 3** | | | | | | | |
| **All** | .732 | .727 | .632 | .801 | .725 | .629 | .799 |  |
| **Site 1** | .836 | .836 | .744 | .896 | .835 | .744 | .896 |  |
| **Site 2** | .625 | .614 | .424 | .752 | .611 | .422 | .749 |  |
|  | Z = 2.50^*^ |  |  |  |  |  |  |  |
| ***Fluid Composite*** | | | | | | | | |
|  | **Year 1 to Year 2** | | | | | | | |
| **All** | .645 | .644 | .545 | .726 | .640 | .540 | .723 |  |
| **Site 1** | .740 | .738 | .621 | .822 | .734 | .617 | .819 |  |
| **Site 2** | .481 | .481 | .294 | .633 | .480 | .293 | .631 |  |
|  | Z = 2.67^**^ |  |  |  |  |  |  |  |
|  | **Year 2 to Year 3** | | | | | | | |
| **All** | .760 | .760 | .673 | .826 | .744 | .637 | .820 |  |
| **Site 1** | .749 | .749 | .619 | .839 | .722 | .550 | .829 |  |
| **Site 2** | .716 | .714 | .556 | .822 | .709 | .550 | .818 |  |
|  | Z = .38 |  |  |  |  |  |  |  |
|  | **Year 1 to Year 3** | | | | | | | |
| **All** | .660 | .660 | .547 | .749 | .628 | .470 | .740 |  |
| **Site 1** | .739 | .738 | .603 | .831 | .681 | .413 | .821 |  |
| **Site 2** | .493 | .488 | .265 | .662 | .477 | .256 | .652 |  |
|  | Z = 2.15^*^ |  |  |  |  |  |  |  |
|  | ***r*** | **ICC (3,1) Consistency** | | | **ICC (3,1) Absolute Agreement** | | |  |
|  |  | **ICC** | **Lower Bound** | **Upper Bound** | **ICC** | **Lower Bound** | **Upper Bound** |  |
| ***Total Composite Score*** | | | | | | | | |
|  | **Year 1 to Year 2** | | | | | | | |
| **All** | .767 | .767 | .695 | .823 | .765 | .693 | .822 |  |
| **Site 1** | .852 | .849 | .775 | .899 | .847 | .773 | .898 |  |
| **Site 2** | .657 | .650 | .503 | .760 | .650 | .503 | .760 |  |
|  | Z = 2.98^**^ |  |  |  |  |  |  |  |
|  | **Year 2 to Year 3** | | | | | | | |
| **All** | .831 | .830 | .765 | .878 | .815 | .728 | .874 |  |
| **Site 1** | .791 | .791 | .679 | .867 | .774 | .638 | .860 |  |
| **Site 2** | .836 | .830 | .726 | 897 | .819 | .701 | .891 |  |
|  | Z = -1.59 |  |  |  |  |  |  |  |
|  | **Year 1 to Year 3** | | | | | | | |
| **All** | .708 | .703 | .602 | .783 | .684 | .559 | .775 |  |
| **Site 1** | .810 | .810 | .706 | .879 | .777 | .597 | .872 |  |
| **Site 2** | .554 | .531 | .318 | .693 | .522 | .310 | .686 |  |
|  | Z = 2.65^**^ |  |  |  |  |  |  |  |

Statistical significance was only calculated for *Z* score transformations comparing Pearson correlations between sites: **p* < .05; ***p* < .01, ****p* < .001

*N*’s for Year 1 to Year 2: *N*_all_ = 163, *N*_site1_ = 83, *N*_site 2_ = 80; *N*’s for Year 2 to Year 3: *N*_all_ = 118, *N*_site1_ = 65, *N*_site 2_ = 53; *N*’s for Year 1 to Year 3: *N*_all_ = 118, *N*_site1_ = 65, *N*_site 2_ = 53

Supplemental Table 5. ICC absolute agreement values marking test-retest reliability of fully normed T scores between 39 participants who identified as Hispanic/Latino (HL) versus 39 age-, sex-, and race-, and education-matched participants who identified as Non-Hispanic/Latino (NHL).

|  |  | **Year 1 to Year 2** | | **Year 2 to Year 3** | | **Year 1 to Year 3** | |
| --- | --- | --- | --- | --- | --- | --- | --- |
|  |  | **ICC** | **95%CI** | **ICC** | **95%CI** | **ICC** | **95%CI** |
| **DCCS** | **NHL** | .394 | .041, .659 | .520 | .161, .759 | .745 | .485, .885 |
|  | **HL** | .116 | -.226, .440 | .315 | -.107, .636 | .201 | -.172, .536 |
|  |  |  |  |  |  |  |  |
| **Flanker** | **NHL** | .265 | -.110, .570 | .001 | -.408, .403 | .527 | .157, .770 |
|  | **HL** | .580 | .277, .775 | .515 | .144, .758 | .426 | .063, .694 |
|  |  |  |  |  |  |  |  |
| **List WM** | **NHL** | .651 | .384, .818 | .735 | .474, .876 | .684 | .376, .856 |
|  | **HL** | .469 | .142, .703 | .481 | .096, .738 | .535 | .186, .765 |
|  |  |  |  |  |  |  |  |
| **Proc Speed** | **NHL** | .706 | .473, .848 | .744 | .492, .880 | .648 | .320, .837 |
|  | **HL** | .587 | .249, .787 | .748 | .460, .887 | .452 | .062, .719 |
|  |  |  |  |  |  |  |  |
| **Pic Mem** | **NHL** | .373 | .013, .645 | .280 | -.074, .591 | .574 | .152, .808 |
|  | **HL** | .299 | -.036, .580 | .401 | .030, .682 | .388 | .021, .672 |
|  |  |  |  |  |  |  |  |
| **Oral Read** | **NHL** | .726 | .504, .859 | .610 | .290, .809 | .634 | .299, .830 |
|  | **HL** | .738 | .526, .864 | .793 | .474, .915 | .773 | .512, .899 |
|  |  |  |  |  |  |  |  |
| **Pic Vocab** | **NHL** | .510 | .191, .732 | .701 | .429, .858 | .685 | .374, .856 |
|  | **HL** | .673 | .421, .829 | .715 | .444, .866 | .652 | .355, .830 |
|  |  |  |  |  |  |  |  |
| **Crystal** | **NHL** | .761 | .555, .879 | .736 | .477, .877 | .722 | .441, .874 |
|  | **HL** | .791 | .610, .894 | .825 | .650, .917 | .731 | .472, .872 |
|  |  |  |  |  |  |  |  |
| **Fluid** | **NHL** | .727 | .503, .860 | .577 | .233, .792 | .864 | .704, .941 |
|  | **HL** | .591 | .311, .778 | .674 | .396, .839 | .515 | .180, .746 |
|  |  |  |  |  |  |  |  |
| **Total** | **NHL** | .821 | .656, .911 | .713 | .440, .865 | .807 | .597, .915 |
|  | **HL** | .793 | .617, .894 | .795 | .565, .906 | .756 | .419, .895 |

“NHL” = non-Hispanic/Latino; “HL” = Hispanic/Latino; “ICC” = intraclass correlation coefficient using a two-way mixed model of absolute agreement; “95%CI” = 95% confidence interval about the ICC estimate; “DCCS” = dimensional change card sorting; “List WM” = list sorting working memory; “Proc Speed” = pattern comparison processing speed; “Pic Mem” = picture sequence memory; “Oral Read” = oral reading; “Pic Vocab” = picture vocabulary; “Crystal” = crystallized cognition composite; “Fluid” = fluid cognition composite; “Total” = total cognition composite

Supplemental Table 6. ICC absolute agreement values marking test-retest reliability of fully normed T scores among youth who were 9- 10- 11- 12- 13- or 14-years-old and older at the time of their first visit.

|  |  | **Year 1 to Year 2** | | **Year 2 to Year 3** | | **Year 1 to Year 3** | |
| --- | --- | --- | --- | --- | --- | --- | --- |
|  |  | **ICC** | **95%CI** | **ICC** | **95%CI** | **ICC** | **95%CI** |
| **DCCS** | **9-year-olds** | .073 | -.273, .753 | .360 | -.085, .682 | .531 | .150, .774 |
|  | **10-year-olds** | .373 | -.005, .659 | .600 | .261, .810 | .606 | .263, .873 |
|  | **11-year-olds** | .683 | .417, .840 | .693 | .398, .859 | .563 | .200, .794 |
|  | **12-year-olds** | .347 | -.112, .680 | .359 | -.114, .711 | .725 | .312, .911 |
|  | **13-year-olds** | .755 | .508, .888 | .760 | .466, .899 | .705 | .353, .884 |
|  | **14-year-olds** | .061 | -.276, .437 | .380 | -.140, .752 | .376 | -.193, .757 |
|  |  |  |  |  |  |  |  |
| **Flanker** | **9-year-olds** | .128 | -.220, .454 | .152 | -.314, .548 | .728 | .446, .878 |
|  | **10-year-olds** | .183 | -.228, .533 | .418 | .023, .705 | .402 | .010, .690 |
|  | **11-year-olds** | .326 | -.027, .613 | .248 | -.165, .595 | .355 | -.097, .680 |
|  | **12-year-olds** | .659 | .319, .849 | .685 | .293, .878 | .675 | .174, .896 |
|  | **13-year-olds** | .297 | -.100, .617 | .385 | -.056, .701 | .157 | -.310, .575 |
|  | **14-year-olds** | .445 | .040, .731 | .567 | -.119, .824 | .602 | .131, .857 |
|  |  |  |  |  |  |  |  |
| **List WM** | **9-year-olds** | .551 | .241, .758 | .449 | .023, .735 | .585 | .222, .805 |
|  | **10-year-olds** | .412 | .046, .683 | .721 | .440, .873 | .294 | -.130, .625 |
|  | **11-year-olds** | .618 | .313, .805 | .627 | .300, .825 | .536 | .121, .786 |
|  | **12-year-olds** | .699 | .380, .869 | .270 | -.184, .652 | .316 | -.264, .737 |
|  | **13-year-olds** | .407 | .006, .693 | .630 | .284, .833 | .540 | .080, .807 |
|  | **14-year-olds** | .385 | -.074, .705 | .713 | .288, .903 | .617 | .161, .863 |
|  |  |  |  |  |  |  |  |
| **Proc Speed** | **9-year-olds** | .818 | .655, .909 | .826 | .620, .926 | .870 | .716, .944 |
|  | **10-year-olds** | .593 | .278, .794 | .691 | .375, .861 | .527 | .020, .794 |
|  | **11-year-olds** | .564 | .254, .770 | .644 | .296, .838 | .401 | -.025, .706 |
|  | **12-year-olds** | .627 | .273, .832 | .754 | .433, .906 | .368 | -.241, .766 |
|  | **13-year-olds** | .442 | .080, .708 | .762 | .489, .899 | .027 | -.461, .493 |
|  | **14-year-olds** | .478 | .072, .752 | .850 | .583, .952 | .355 | -.255, .751 |
|  |  |  |  |  |  |  |  |
| **Pic Mem** | **9-year-olds** | .432 | .108, .678 | .790 | .081, .757 | .593 | .246, .807 |
|  | **10-year-olds** | .024 | -.299, .372 | .170 | -.275, .549 | .240 | -.155, .583 |
|  | **11-year-olds** | .343 | -.026, .631 | .198 | -.230, .564 | .194 | -.220, .561 |
|  | **12-year-olds** | .602 | .244, .819 | .458 | -.035, .773 | .444 | -.062, .791 |
|  | **13-year-olds** | .404 | .035, .684 | .630 | .262, .836 | .438 | -.026, .750 |
|  | **14-year-olds** | .330 | -.091, .661 | .706 | .298, .899 | .677 | .247, .888 |
|  |  |  |  |  |  |  |  |
| **Oral Read** | **9-year-olds** | .769 | .570, .883 | .829 | .625, .927 | .821 | .616, .921 |
|  | **10-year-olds** | .714 | .460, .861 | .722 | .391, .880 | .556 | .184, .798 |
|  | **11-year-olds** | .597 | .291, .791 | .376 | -.022, .678 | .319 | -.122, .654 |
|  | **12-year-olds** | .622 | .258, .831 | .454 | .006, .761 | .770 | .373, .928 |
|  | **13-year-olds** | .621 | .285, .820 | .701 | .391, .869 | .454 | -.050, .770 |
|  | **14-year-olds** | .897 | .757, .958 | .627 | .169, .867 | .700 | .292, .896 |
|  |  |  |  |  |  |  |  |
|  |  |  |  |  |  |  |  |
|  |  |  |  |  |  |  |  |
|  |  | **Year 1 to Year 2** | | **Year 2 to Year 3** | | **Year 1 to Year 3** | |
|  |  | **ICC** | **95%CI** | **ICC** | **95%CI** | **ICC** | **95%CI** |
| **Pic Vocab** | **9-year-olds** | .695 | .423, .847 | .584 | .224, .806 | .293 | -.125, .627 |
|  | **10-year-olds** | .719 | .469, .863 | .650 | .317, .839 | .802 | .590, .911 |
|  | **11-year-olds** | .519 | .186, .745 | .642 | .319, .833 | .759 | .473, .897 |
|  | **12-year-olds** | .529 | .125, .783 | .607 | .168, .843 | .723 | .297, .911 |
|  | **13-year-olds** | .309 | -.111, .631 | .767 | .499, .901 | .351 | -.164, .708 |
|  | **14-year-olds** | .777 | .515, .906 | .540 | .008, .833 | .312 | -.205, .714 |
|  |  |  |  |  |  |  |  |
| **Crystal** | **9-year-olds** | .824 | .597, .920 | .873 | .719, .945 | .775 | .539, .898 |
|  | **10-year-olds** | .741 | .506, .874 | .756 | .512, .888 | .700 | .378, .865 |
|  | **11-year-olds** | .681 | .421, .839 | .619 | .286, .821 | .702 | .396, .867 |
|  | **12-year-olds** | .727 | .434, .882 | .566 | .108, .824 | .833 | .534, .948 |
|  | **13-year-olds** | .550 | .199, .776 | .803 | .564, .917 | .377 | -.118, .719 |
|  | **14-year-olds** | .943 | .862, .977 | .729 | .333, .905 | .677 | .237, .885 |
|  |  |  |  |  |  |  |  |
| **Fluid** | **9-year-olds** | .681 | .431, .834 | .619 | .270, .823 | .847 | .674, .932 |
|  | **10-year-olds** | .549 | .215, .769 | .673 | .376, .846 | .554 | .093, .801 |
|  | **11-year-olds** | .809 | .557, .915 | .646 | .258, .844 | .581 | .014, .835 |
|  | **12-year-olds** | .731 | .444, .883 | .638 | .087, .871 | .616 | .093, .873 |
|  | **13-year-olds** | .196 | -.235, .554 | .859 | .681, .942 | .250 | -.267, .648 |
|  | **14-year-olds** | .481 | .090, .863 | .830 | .547, .942 | .680 | .246, .885 |
|  |  |  |  |  |  |  |  |
| **Total** | **9-year-olds** | .857 | .724, .929 | .783 | .545, .904 | .884 | .749, .949 |
|  | **10-year-olds** | .697 | .419, .853 | .749 | .477, .887 | .625 | .120, .846 |
|  | **11-year-olds** | .815 | .599, .915 | .622 | .190, .837 | .570 | .072, .820 |
|  | **12-year-olds** | .526 | .678, .942 | .644 | .170, .866 | .801 | .460, .938 |
|  | **13-year-olds** | .353 | -.063, .660 | .881 | .723, .951 | .281 | -.228, .665 |
|  | **14-year-olds** | .786 | .532, .910 | .857 | .612, .952 | .640 | .175, .870 |

*N*’s for Year 1 to Year 2: *N*_9_ = 30, *N*_10_ = 26, *N*_11_ = 28, *N*_12_ = 20, *N*_13_ = 23, *N*_14+_ = 20; *N*’s for Year 2 to Year 3 and for Year 1 to Year 3: *N*_9_ = 22, *N*_10_ = 23, *N*_11_ = 21, *N*_12_ = 12, *N*_13_ = 16, *N*_14+_ = 13

“ICC” = intraclass correlation coefficient using a two-way mixed model of absolute agreement; “95%CI” = 95% confidence interval about the ICC estimate; “DCCS” = dimensional change card sorting; “List WM” = list sorting working memory; “Proc Speed” = pattern comparison processing speed; “Pic Mem” = picture sequence memory; “Oral Read” = oral reading; “Pic Vocab” = picture vocabulary; “Crystal” = crystallized cognition composite; “Fluid” = fluid cognition composite; “Total” = total cognition composite

Supplemental Table 7. ICC absolute agreement values marking test-retest reliability of fully normed T scores between males versus females.

|  |  | **Year 1 to Year 2** | | **Year 2 to Year 3** | | **Year 1 to Year 3** | |
| --- | --- | --- | --- | --- | --- | --- | --- |
|  |  | **ICC** | **95%CI** | **ICC** | **95%CI** | **ICC** | **95%CI** |
| **DCCS** | **Male** | .454 | .256, .615 | .593 | .410, .729 | .603 | .418, .740 |
|  | **Female** | .370 | .159, .552 | .604 | .392, .756 | .553 | .310, .727 |
|  |  |  |  |  |  |  |  |
| **Flanker** | **Male** | .280 | .065, .472 | .428 | .210, .606 | .635 | .459, .763 |
|  | **Female** | .648 | .134, .532 | .387 | .117, .604 | .337 | .061, .567 |
|  |  |  |  |  |  |  |  |
| **List WM** | **Male** | .584 | .416, .713 | .573 | .388, .714 | .505 | .289, .670 |
|  | **Female** | .482 | .282, .641 | .527 | .287, .704 | .477 | .217, .673 |
|  |  |  |  |  |  |  |  |
| **Proc Speed** | **Male** | .664 | .502, .777 | .709 | .562, .812 | .439 | .209, .622 |
|  | **Female** | .517 | .318, .670 | .808 | .675, .889 | .506 | .240, .698 |
|  |  |  |  |  |  |  |  |
| **Pic Mem** | **Male** | .352 | .136, .534 | .470 | .258, .638 | .495 | .253, .673 |
|  | **Female** | .328 | .113, .516 | .280 | -.003, .521 | .352 | .079, .579 |
|  |  |  |  |  |  |  |  |
| **Oral Read** | **Male** | .706 | .573, .802 | .637 | .469, .760 | .664 | .498, .783 |
|  | **Female** | .729 | .600, .822 | .666 | .471, .799 | .607 | .382, .764 |
|  |  |  |  |  |  |  |  |
| **Pic Vocab** | **Male** | .678 | .537, .782 | .596 | .414, .732 | .591 | .403, .731 |
|  | **Female** | .519 | .327, .670 | .683 | .496, .809 | .507 | .255, .694 |
|  |  |  |  |  |  |  |  |
| **Crystal** | **Male** | .804 | .708, .970 | .746 | .618, .836 | .761 | .833, .849 |
|  | **Female** | .687 | .543, .792 | .740 | .584, .843 | .545 | .307, .718 |
|  |  |  |  |  |  |  |  |
| **Fluid** | **Male** | .683 | .536, .789 | .712 | .565, .814 | .662 | .455, .793 |
|  | **Female** | .486 | .286, .644 | .694 | .517, .814 | .506 | .262, .689 |
|  |  |  |  |  |  |  |  |
| **Total** | **Male** | .795 | .682, .868 | .757 | .629, .844 | .708 | .515, .825 |
|  | **Female** | .647 | .489, .764 | .763 | .609, .860 | .505 | .260, .690 |

*N*’s for Year 1 to Year 2: *N*_Male_ = 77, *N*_Female_ = 71; *N*’s for Year 2 to Year 3 and for Year 1 to Year 3: *N*_Male_ = 62, *N*_Female_ = 45

“ICC” = intraclass correlation coefficient using a two-way mixed model of absolute agreement; “95%CI” = 95% confidence interval about the ICC estimate; “DCCS” = dimensional change card sorting; “List WM” = list sorting working memory; “Proc Speed” = pattern comparison processing speed; “Pic Mem” = picture sequence memory; “Oral Read” = oral reading; “Pic Vocab” = picture vocabulary; “Crystal” = crystallized cognition composite; “Fluid” = fluid cognition composite; “Total” = total cognition composite

**Figure S1 (caption on next page)**


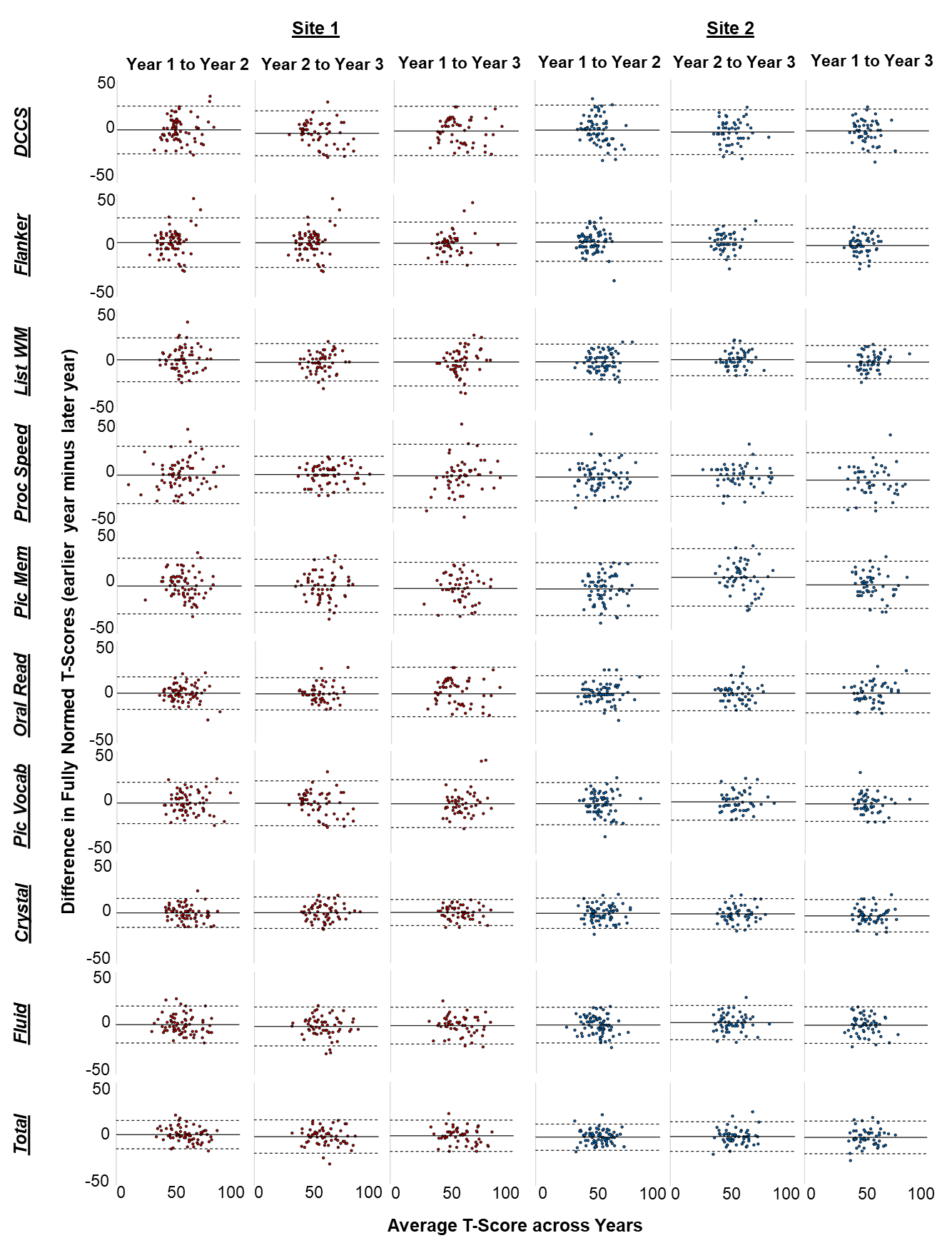


*Figure S1.* Bland-Altman plots depicting patterns of deviation in fully normed T-scores over time, separately for each site. The solid black line in each plot is the bias (i.e., the mean difference between years); dashed lines are the upper and lower limits of agreement (bias ±1.96**SD*). Site 1: *n_year1_* = 100, *n_year2_* = 83, *n_year3_* = 65. Site 2: *n_year1_* = 92, *n_year2_* = 79, *n_year3_* = 53. DCCS = Dimensional Change Card Sorting; Flanker = Flanker Test of Inhibitory Control and Attention; List WM = List Sorting Working Memory; Proc Speed = Pattern Comparison Processing Speed; Pic Mem = Picture Sequence Memory; Oral Read = Oral Reading; Pic Vocab = Picture Vocabulary; Crystal = Crystalized Cognition composite score; Fluid = Fluid Cognition composite score; Total = Total Cognition composite score

**Figure S2 (caption on next page)**


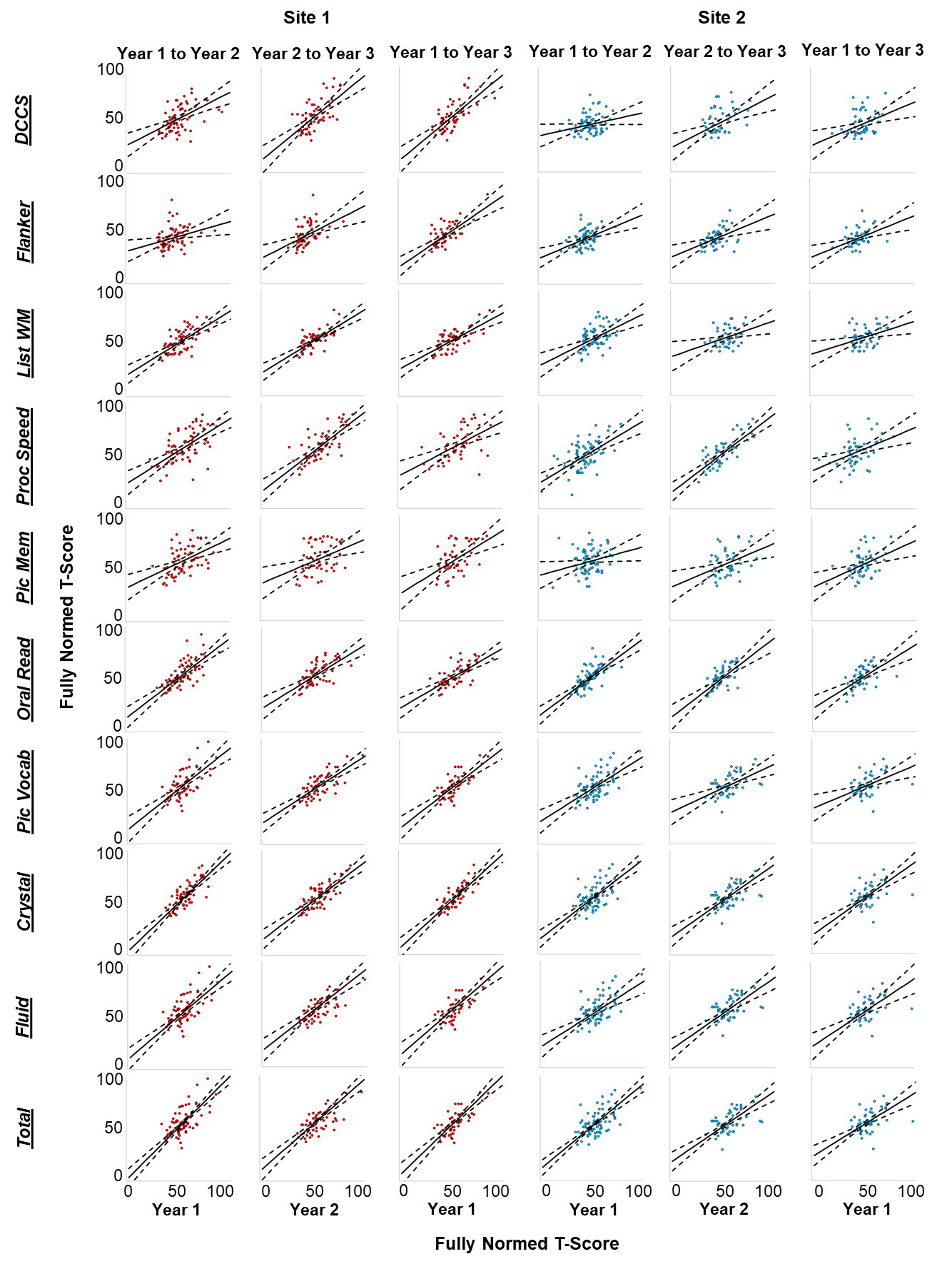


*Figure S2*. Scatterplots depicting the correlations between fully normed T-scores for each NIH-TB Cognitive Battery subtest, separately by study site, for each of the three tested intervals. Solid black lines indicate the line of best fit (i.e., the Pearson correlation) through the data. Dashed black lines show the upper and lower bounds of the 95% confidence interval around the line of best fit. DCCS = Dimensional Change Card Sorting; Flanker = Flanker Test of Inhibitory Control and Attention; List WM = List Sorting Working Memory; Proc Speed = Pattern Comparison Processing Speed; Pic Mem = Picture Sequence Memory; Oral Read = Oral Reading; Pic Vocab = Picture Vocabulary; Crystal = Crystalized Cognition composite score; Fluid = Fluid Cognition composite score; Total = Total Cognition composite score
